# Sociocognitive determinants of adherence to standard precautions by the nursing staff in a regional hospital: a mixed-methods study

**DOI:** 10.1590/1980-220X-REEUSP-2025-0335en

**Published:** 2026-03-23

**Authors:** Junaiza Rodrigues Lima Nunes, Cleydson Wendel Nunes de Souza, Allyne Brena Coimbra Azevedo, Cristianne Teixeira Carneiro, Mychelangela de Assis Brito, Maria Augusta Rocha Bezerra, Ruth Cardoso Rocha

**Affiliations:** 1 Universidade Federal do Piauí, Floriano, PI, Brazil.

**Keywords:** Occupational Health, Universal Precautions, Nursing

## Abstract

**Objective:**

To analyze the sociocognitive determinants of adherence to standard precautions by the nursing staff in a Brazilian regional hospital.

**Method:**

Mixed methodology study, explanatory sequential design, conducted between January 2023 and March 2024, with 230 professionals. The Brazilian version of *Standard Precautions Questionnaire* and semi-structured interviews were used. The quantitative analysis employed the Mann-Whitney test, and the qualitative analysis, content analysis.

**Results:**

The overall mean sociocognitive adherence score was 3.90 (SD = 0.48), with the highest score in the “Attitude” factor (4.60; SD = 0.48) and the lowest in “Organizational Constraints” (2.95; SD = 1.17). Significant differences were identified between professional categories in “Social Influence” (nurses: 4.72; technicians: 4.79; p = 0.001) and “Organization” (nurses: 4.60; technicians: 4.49; p = 0.033). The integration of the findings demonstrated convergence between quantitative and qualitative data, showing that adherence, although satisfactory, is influenced by work-related factors.

**Conclusions:**

Technicians demonstrated greater social influence, while nurses identified more organizational barriers. Peer example encourages adherence, despite the persistent fear of reprimands for non-compliance with best practices.

## INTRODUCTION

Nursing professionals represent a significant portion of the workforce in hospital settings^(1)^, being exposed to different occupational risks of a biological, chemical, physical and ergonomic nature^(2)^. In this context, biological risks represent one of the most commonly faced threats^(3)^ involving contact with blood, tissues, or other potentially infectious bodily fluids^(2)^.

To mitigate these risks and prevent injuries, it is essential to provide access to and encourage adherence^(4)^ to biosafety measures, especially Standard Precautions (SP), which aim to limit the transmission of infectious agents in healthcare settings, such as hand hygiene, the use of Personal Protective Equipment (PPE) – gloves, aprons, goggles and masks – and the safe handling of sharps and contaminated materials^(5)^.

Although nursing professionals show greater adherence to SPs, compared to other healthcare professionals^(6)^, the rates remain below the ideal^(7)^. It is observed that up to 68.3% of nurses have unsatisfactory knowledge about SPs and 73.3% do not systematically adopt them^(8)^. Reasons frequently associated with this non-adherence include work disorganization, work overload, double shifts, emergency situations, lack of materials, and individual factors such as forgetfulness and low professional qualifications^(9)^.

This context has contributed to the increased incidence of occupational injuries among nursing professionals^(10)^, whose prevalence, throughout their careers, has reached more than half of nurses^(11)^. Therefore, it becomes essential to identify the underlying reasons for non-compliance with SPs^(4)^, aiming to fill gaps identified in previous studies^(12,13)^ and to deepen the understanding of the determinants that influence this practice.

Among the factors influencing human behavior and decision-making among nursing professionals, sociocognitive determinants stand out, such as knowledge, motivation, intention, expectations, and perceptions^(13)^. These elements play a fundamental role in how these professionals adhere to SP practices, especially in contexts characterized by high complexity, dynamism, and frequently urgent situations^(14)^.

However, there is a gap in the scientific literature regarding studies that specifically address the sociocognitive determinants and their relationship with the behavior of nursing professionals in this setting. This finding emphasizes the need for greater understanding, recognition, and critical evaluation of these factors^(13)^. Incorporating mixed data into the analysis contributes to a more in-depth interpretation, allowing for a more comprehensive analysis of this phenomenon^(15)^. Given the above, the objective was to analyze the sociocognitive determinants influencing adherence to standard precautions by the nursing staff in a Brazilian regional hospital.

## METHOD

### Design of Study

The study used a mixed-methods approach, with a sequential explanatory design (QUAN→qual), in which the quantitative phase preceded and guided the qualitative analysis. Qualitative findings were used to further interpret the quantitative results^(16)^. The quantitative phase was cross-sectional, while the qualitative phase was analytical in nature. To ensure methodological rigor, the *Mixed Methods Appraisal Tool* (MMAT) was applied. The investigation sought to answer the question: what sociocognitive determinants influence adherence to Standard Precautions by the nursing staff in a Brazilian regional hospital?

### Local

The research was conducted in a regional hospital located in the interior of the state of Piauí, Brazil. This is a general hospital, which provides surgical, clinical, obstetric, pediatric, and other services, whose main function is to provide healthcare. In addition, the institution develops secondary activities, such as diagnostic support, emergency care, medication dispensing, immunization, health promotion, disease prevention, and the provision of medium and high complexity care. In 2023, the institution had 745 beds, divided into the following sectors: Surgical Suite, Central Sterile Supply Department, Surgical Clinic, Medical Clinic, Obstetrics, Pediatrics, Emergency Room, and Intensive Care Unit.

### Population and Selection Criteria

The study population consisted of 377 nursing professionals working at the hospital, comprising 112 nurses and 265 nursing technicians, according to data provided by the institution. The inclusion criteria were established as follows: being a professional member of the nursing team working at the regional hospital for at least a year and having direct contact with the patient. Those who were absent due to vacation or medical leave were excluded.

### Sample Definition

The sample was selected by convenience, and participants were recruited in person and individually, in the different sectors of the institution. For the quantitative data collection, a sample size calculation was performed to ensure a significant number of participants, adopting a 95% confidence level and a 5% margin of error, resulting in an estimated minimum sample of 191 nursing professionals. However, the final sample was expanded, totaling 230 participants in the quantitative phase.

For qualitative data collection, participant selection was performed using purposeful sampling, considering criteria of relevance for a deeper understanding of the phenomenon under investigation. The aim was to include a diversity of professional experiences, positions, and areas of expertise, to ensure proportionality between nurses and nursing technicians and to encompass different perspectives on SP. The sample was closed due to theoretical saturation, reached when new interviews ceased to add substantially new information to the analytical categories, resulting in a total of 15 participants in this phase.

### Data Collection

Data collection took place between January 2023 and March 2024. In the quantitative phase, sociodemographic data (sex; age; ethnicity; income) and professional training data (training category; time since graduation; sector of activity; work shift; number of jobs; job satisfaction; time working in the profession; time working at the institution; and work-related illness) were collected.

Subsequently, the Brazilian version of the *Standard Precautions Questionnaire* (SPQ) was used, originally developed and validated in France, aimed at assessing the sociocognitive determinants of adherence to SPs, such as attitudes, behaviors, personal and organizational limitations^(17)^. The SPQ underwent cultural adaptation and semantic validation for Brazilian Portuguese, demonstrating satisfactory psychometric properties, including construct validity and reliability^(18)^. The instrument consists of 24 items distributed across seven factors: 1 – Interpersonal behavior (2 items), 2 – Organizational restrictions (4 items), 3 – Intention to follow SPs (4 items), 4 – Social influence (4 items), 5 – Attitudes towards the SP (3 items), 6 – Organization (3 items), and 7 – Individual restrictions (4 items)^(18,19)^.

The qualitative phase was conducted using a semi-structured script, designed to explore the perceptions of nursing professionals regarding the application of the SPs. The interviews, lasting an average of 10 minutes, were conducted individually in a private room, audio-recorded, and transcribed in full. Before this stage began, the script was validated with nurses from another service, who were not included in the final sample. This allowed for adjustments to the instrument and improvement of the interview technique. It is also emphasized that the three research assistants, responsible for data collection, underwent prior training, guided by the principal investigator.

### Data Analysis and Treatment

The quantitative data were organized in Microsoft Excel® 2016 using double entry and subsequently analyzed using SPSS® software, version 20.0. Descriptive statistics was used, including measures of central tendency and dispersion, as well as the Shapiro-Wilk test to verify the adherence of the variables to a normal distribution. Given the lack of normality in some of the data, the non-parametric Mann-Whitney test was applied to compare the instrument scores across professional categories^(20)^. A 95% confidence interval and a significance level of p ≤ 0.05 were adopted.

Qualitative data were organized in three stages: pre-analysis, in which the material was organized to make it functional and systematize the initial ideas; survey/categorization of the material, in which the material was examined, and the categories and units of recording and context were defined. Finally, in the interpretation of the results, an analytical description was carried out, in which the material was subjected to in-depth study, guided by hypotheses and theoretical frameworks^(21)^. Data integration was carried out by connecting the quantitative and qualitative results, seeking convergences and divergences and deepening the analysis of the object of study.

### Ethical Aspects

This study followed the guidelines of the National Health Council for research with human beings, according to Resolutions No. 466/2012, 510/2016 and 580/2018, and was approved by the Research Ethics Committee of the Universidade Federal do Piauí, under opinions No. 5,834,174 and No. 6,086,132. The participants’ identities were preserved through the assignment of alphanumeric codes (P1, P2, P3…), and all signed the Free Informed Consent Form.

## RESULTS

The study included 230 nursing professionals from the regional hospital, aged between 19 and 70 years. The majority were female (81.7%), predominantly young people between 19 and 40 years old (80.0%). Regarding ethnicity, 54.4% self-identified themselves as Black. Nursing technicians predominated (66.1%), with time since training completion between six and ten years (30.4%) and working in all three shifts (56.1%). Most had only one job (68.7%) and a salary between R$1,000.00 and R$3,000.00 (76.5%), in addition to high job satisfaction (86.5%). Regarding length of employment, the most common interval was one to five years, both in the profession (43.5%) and in the institution (47.0%). Finally, 75.3% reported having no occupational diseases. The data described are detailed in Table 1.

**Table 1:** Socioeconomic and professional characterization of nursing professionals working in a regional hospital – PI, Brazil, 2023–2024.

Variables	N	%	Mean ± SD
Sex			
Female	188	81.7	
Male	42	18.3	
Age			33.88 ± 9.75
19–40	184	80.0	
41–70	46	20.0	
Ethnicity			
Black	125	54.4	
Brown	76	33.0	
White	28	12.2	
Indigenous	1	0.4	
Professional category			
Nursing technician	152	66.1	
Nurse	78	33.9	
Time since graduation (years)			
1–5	95	41.3	
6–10	70	30.4	
>10	64	27.9	
Not informed	1	0.4	
Area of activity			
Operating Suite	51	22.2	
Obstetrics	37	16.1	
Emergency Room	31	13.5	
Internal Medicine	29	12.6	
Intensive Care Unit	27	11.7	
Surgical Clinic	22	9.6	
Central Sterile Supply Department	21	9.1	
Pediatrics	12	5.2	
Work shift			
Morning/Afternoon/Evening	129	56.1	
Morning	33	14.3	
Morning/Evening	22	9.6	
Morning/Afternoon	15	6.5	
Evening	14	6.2	
Afternoon	10	4.3	
Afternoon/Evening	6	2.6	
Not informed	1	0.4	
Number of jobs			
One	158	68.7	
Two	61	26.5	
Three or more	9	3.9	
Not informed	2	0.9	
Income (minimum wages)			
1–3	176	76.5	
3–6	48	20.9	
> 6	5	2.2	
Not informed	1	0.4	
Job satisfaction			
Yes	199	86.5	
No	31	13.5	
Length of time working in the profession (years)		
< 1	46	20.0	
1–5	100	43.5	
6–10	38	16.5	
>10	45	19.6	
Not informed	1	0.4	
Time working in the institution (years)			
< 1	58	25.2	
1–5	108	47.0	
6–10	36	15.7	
>10	28	12.1	
Work-related illness			
Yes	47	20.4	
No	173	75.3	
I don’t know	10	4.3	

In Table 2, the results obtained from applying the questionnaire *Standard Precautions Questionnaire* (SPQ) are presented. In the “Attitude” factor (part 1), the majority of participants strongly agreed that SPs are an effective way to prevent hospital infections (68.3%), as well as to protect patients (67.4%) and professionals (67.4%). Regarding the “Social Influence” factor, 36.1% partially believed that colleagues valued SPs. However, 48.7% reported a risk of being warned by superiors when they do not follow the guidelines, which is the most prevalent percentage in this area. Regarding the “Organization” factor, three items stood out with a prevalence above 60%: 65.7% reported the total effectiveness of the availability of materials; 60.4% indicated that being trained favors adherence; and 61.3% reinforced the importance of frequent training.

**Table 2:** Distribution, percentage, mean, and standard deviation, according to items from the Brazilian version of the *Standard Precautions Questionnaire*, answered by nursing professionals – PI, Brazil, 2023–2024.

Items	Ineffective n (%)	A little effective n (%)	More or less effective n (%)	Partially effective n (%)	Fully effective n (%)	Did not answer n (%)	Mean	Standard deviation
**Part 1 – Attitude**
Standard precautions are effective measures to reduce hospital-acquired infections.	5 2.2%	–	8 3.5%	59 25.7%	157 68.3%	1 0.4%	4.59	0.76
If I follow standard precautions, I will protect my patients from infection.	–	–	9 3.9%	65 28.3%	155 67.4%	1 0.4%	4.64	0.56
Following standard precautions will protect me from infection.	1 0.4%	2 0.9%	11 4.8%	62 27%	154 67.0%	–	4.59	0.66
**Part 2 – Social Influence**								
Most of my coworkers think it’s important to follow standard precautions.	4 1.7%	14 6.1%	70 30.4%	83 36.1%	57 24.8%	2 0.9%	3.78	0.97
I risk receiving warnings from my superiors if I don’t follow standard precautions.	5 2.2%	8 3.5%	22 9.6%	83 36.1%	112 48.7%	–	4.25	0.92
I risk receiving warnings from the nurses and assistants responsible for hygiene if I do not follow standard precautions.	8 3.5%	16 7.0%	27 11.7%	87 37.8%	90 39.1%	1 0.4%	4.03	1.06
I risk receiving warnings from doctors if I don’t follow standard precautions.	19 8.3%	19 8.3%	38 16.5%	69 30.0%	83 36.1%	2 0.9%	3.80	1.26
**Part 3 – Organization**								
Having materials (quality, availability, and accessibility) in all workplaces.	1 0.4%	4 1.7%	19 8.3%	54 23.5%	151 65.7%	1 0.4%	4.53	0.76
Being proficient in standard precautions	1 0.4%	2 0.9%	16 7.0%	70 30.4%	139 60.4%	2 0.9%	4.52	0.71
Having training regarding standard precautions	2 0.9%	2 0.9%	10 4.3%	74 32.2%	141 61.3%	1 0.4%	4.53	0.70
**Part 4 – Interpersonal Behavior**								
When a medical professional demonstrates exemplary behavior with regard to standard precautions	3 1.3%	14 6.1%	28 12.2%	68 29.6%	116 50.4%	1 0.4%	4.23	0.97
When my colleagues at work demonstrate exemplary behavior with regard to standard precautions.	8 3.5%	7 3.0%	30 13.0%	76 33.0%	108 47.0%	1 0.4%	4.18	1.01
**Items**	**It is not an obstacle n (%)**	**A little bit of an obstacle n (%)**	**More or less obstacle n (%)**	**Partiallyan obstacle n (%)**	**It’s an obstaclen (%)**	**Did not answern (%)**	**Mean**	**Standard deviation**
**Part 5 – Organizational constraints**								
Unexpected situations that may hinder the performance of my work (urgency, requests from colleagues, new task to complete).	44 19.1%	16 7.0%	60 26.1%	52 22.6%	56 24.3%	2 0.9%	3.28	1.42
Lack of time.	89 38.7%	32 13.9%	36 15.7%	30 13.0%	41 17.8%	2 0.9%	2.60	1.56
Higher workload than usual.	56 24.3%	31 13.5%	52 22.6%	30 13.0%	58 25.2%	3 1.3%	3.05	1.54
Complexity of standard precaution measures.	66 28.7%	32 13.9%	50 21.7%	33 14.3%	46 20.0%	3 1.3%	2.87	1.53
**Part 6 – Individual Restrictions**								
Lack of knowledge about standard precautions.	55 23.9%	21 9.1%	33 14.3%	27 11.7%	91 39.6%	3 1.3%	3.37	1.65
Routine, habits, and work team.	81 35.2%	22 9.6%	40 17.4%	28 12.2%	57 24.8%	2 0.9%	2.84	1.63
Personal beliefs related to standard precautions.	88 38.3%	21 9.1%	42 18.3%	24 10.4%	52 22.6%	3 1.3%	2.73	1.64
Problems related to the material (quality, availability and accessibility).	35 15.2%	15 6.5%	37 16.1%	44 19.1%	97 42.2%	2 0.9%	3.69	1.47
**Items**	**Nevern (%)**	**Rarely n (%)**	**Sometimesn (%)**	**Often n (%)**	**Alwaysn (%)**	**Did not answern (%)**	**Mean**	**Standard deviation**
**Part 7 – Intention to follow standard precautions**								
Even when the patient is difficult.	3 1.3%	6 2.6%	24 10.4%	29 12.6%	167 72.6%	1 0.4%	4.53	0.88
Even when there is little time.	2 0.9%	11 4.8%	25 10.9%	39 17.0%	152 66.1%	1 0.4%	4.43	0.93
Even when my hands are sore or injured.	18 7.8%	7 3.0%	22 9.6%	44 19.1%	139 60.4%	–	4.21	1.21
Even in an emergency situation	5 2.2%	10 4.3%	21 9.1%	26 11.3%	167 72.6%	1 0.4%	4.49	0.98

In the “Interpersonal Behavior” category, 50.4% indicated that exemplary physician behavior facilitates adherence, and 47.0% emphasized the positive influence of colleagues. With respect to “Organizational Constraints”, the main obstacle was “unexpected situations” (24.3%), while “lack of time” was not considered an obstacle by 38.7%. In the “Individual Restrictions” category, the main barrier was inadequate materials (42.2%), while personal beliefs were minimized (38.3% stated they were not obstacles). Finally, in “Intention,” the majority always followed the SPs, especially in difficult situations (72.6%).

The SPQ Scale showed an overall average of 3.90 (SD = 0.48). The “Attitude” factor obtained the highest score (4.60; SD: 0.48), while the “Organizational restrictions” factor registered the lowest average (2.95; SD: 1.17). In the comparison by professional category (Table 3), nursing technicians presented a significantly higher score in “Social Influence” (4.09) compared to nurses (3.72; p = 0.001), while nurses scored higher on the “Organization” factor (4.60) compared to technicians (4.49; p = 0.033). For the factors “Attitude,” “Interpersonal behavior,” “Organizational restrictions,” “Individual restrictions,” and “Intention to follow standard precautions,” no statistically relevant differences were observed within the groups.

**Table 3:** Score of sociocognitive determinants related to the factors of the Brazilian version of *Standard Precautions Questionnaire* by professional category of nursing professionals – PI, Brazil, 2023–2024.

Factors	Professional categories		p-value*
	Nurse	Nursing technician	
Attitude	4.64	4.58	0.293
Social influence	3.72	4.09	**0.001**
Organization	4.60	4.49	**0.033**
Interpersonal behavior	4.07	4.27	0.331
Organizational restrictions	3.06	2.89	0.250
Individual restrictions	3.14	3.17	0.947
Intention to follow standard precautions.	4.42	4.41	0.870

*Mann-Whitney test.

Regarding the qualitative analysis of the study, the content of the interviews allowed for the establishment of three analytical categories: Understanding of standard precautions by nursing professionals, Use of standard precautions by the nursing team, and Factors that hinder the use of standard precautions by the nursing team. These categories were incorporated into the quantitative results, with the extraction of significant statements to better represent them, as described in *Joint display*, shown in Chart 1.

**Chart 1:** Joint display integrating quantitative results and participant statements – PI, Brazil, 2023–2024.

Subcategory	Quantitative results (n = 230)	Qualitative results (n = 15)
**Category 1 – Understanding of standard precautions by nursing professionals**		
Understanding the concept of SPs.	68.3% indicated that SPs are effective in reducing hospital-acquired infections. 39.6% indicated that a lack of knowledge about SPs is an obstacle. 38.3% indicated that personal beliefs related to SPs are not an obstacle to adherence. 60.4% indicated that being trained in relation to SPs is totally effective in adherence. 61.3% indicated that being trained in relation to SPs is totally effective in adherence.	*Precaution comes from taking precautions, from prophylaxis, prevention, and standard means that it is a ready-made method to be followed. Therefore, the objective of standard precautions is to standardize procedures to be followed by all healthcare professionals, with the aim of preventing or even controlling the transmission of microorganisms in the workplace, especially in the hospital setting.* (P13)
Information regarding the main SP measurements used.	65.7% indicated that having materials (quality, availability, and accessibility) in all workplaces is effective. 42.2% indicated that problems related to materials (quality, availability, and accessibility) are obstacles to adopting SPs.	*Personal protective equipment, goggles, masks, proper handwashing, sanitizing critical environments such as operating room and equipment, and disposing of sharps in the correct place all contribute to preventing potential workplace accidents and/or contamination*. (P1)
The perception that the use of SPs prevents accidents and protects both the professional and the patient from diseases.	67.4% indicated that following SPs measures will protect patients from infection. 67.0% indicated that following SPs measures will protect patients from infection.	*They [SPs] prevent problems or some type of infection both from the patient to the professional and from the professional to the patient*. (P1)
**Category 2 – Use of standard precautions by the nursing staff**		
When should SPs be used?	72.6% indicated that they always intend to follow the SPs, even when the patient is difficult. 66.1% indicated that they always intend to follow the SPs, even when there is little time. 60.4% indicated that they always intend to follow the SPs, even when their hands are sore or injured. 72.6% indicated that they always intend to follow the SPs, even in emergency situations. 38.7% indicated that lack of time is not an obstacle to adhering to the SPs.	*[…] it should always be used in assistance between one patient and another […] will there be exceptions? Yes! Depending on the case, but it is recommended that they always be used*. (P13) *[…] in case of an emergency, let’s say there’s a cardiorespiratory arrest: you’re performing a simple procedure and need to resuscitate someone, but there’s no time to get fully equipped with personal protective equipment, so we use what’s already nearby*. (P12)
**Category 3 – Factors that interfere with the use of standard precautions by the nursing staff**		
Negligence, forgetfulness, lack of care, carelessness, and allergy.	42.2% indicated that problems related to materials (quality, availability, and accessibility) are obstacles to the adherence to SPs. 25.2% indicated that a higher than usual workload is an obstacle to adherence to SPs.	*Either way, it’s negligence: you have to watch yourself all the time to always be within the standards.* (P7) *Even knowing its importance, sometimes I don’t use it because I don’t have time; sometimes, it relaxes me […] you end up trusting it, even knowing that “you can’t judge a book by its cover”; and sometimes, I don’t use it that much because I’m allergic to latex*. (P5) *When PPE is not provided by the institution, when there is a shortage, or when it has not been restocked […].* (P13) *Do you want me to be honest? Well, to be quite honest, when I don’t use standard precautions it’s purely out of carelessness, even knowing that they protect both professionals and patients.* (P15)
Materials unavailability.	65.7% indicated that having materials (quality, availability, and accessibility) in all workplaces is fully effective in ensuring adherence to SPs.	*[…] we don’t stop using something just because we want to, we only stop using it when we simply don’t have it. Lack is rare, but sometimes it happens, and when it does, we improvise*. (P1)
Collective influence.	36.1% indicated that it is partially effective, when most coworkers think it is important to follow the SPs. 50.4% indicated that it is fully effective when the medical professional exhibits exemplary behavior regarding SPs. 47.0% indicated that it is fully effective when coworkers have exemplary behavior regarding SPs.	*[…] because when I’m using my PPE, I’m safe: I won’t catch it, much less transmit it. If everyone used precautionary measures, it would be more beneficial, but not everyone does*. (P9)

**Note:** SP = standard precautions.

The integration of the results shows strong convergence between the dimensions evaluated, especially in the recognition of the importance of SPs and in the perception of their effectiveness in preventing infections. This aspect is corroborated both by the high scores in “Attitude,” in the quantitative component, and by the reports highlighting the preventive nature and mutual protection provided by the measures. Furthermore, convergence was observed in the identification of organizational barriers, especially regarding the availability and suitability of materials, quantitatively identified as the main obstacle and qualitatively reiterated by mentioning the lack of PPE and the need for improvisation. Collective influence also proved consistent across methods: although quantitative analysis indicated moderate social influence, the discourses reinforced that the exemplary behavior of colleagues and doctors favors adherence.

Conversely, subtle divergences emerged regarding the effective use of SPs: while quantitative data suggested a high intention to adhere, even in adverse situations, qualitative testimonies revealed situations of negligence, forgetfulness, and improvisation, indicating a mismatch between declared intention and daily practice. Overall, the convergence of findings supports the idea that knowledge, resource availability, and the collective environment were central determinants of adherence, while the divergences highlighted the need for institutional strategies aimed at reducing practical and behavioral barriers.

## DISCUSSION

The analysis of the sociocognitive determinants of the nursing staff at a Brazilian regional hospital revealed important advances in the knowledge and stated intention of nurses and nursing technicians regarding adherence to SPs, especially concerning the recognition of their effectiveness in preventing hospital infections. However, this positive perception contrasts with the practical barriers identified, particularly with regard to organizational restrictions and the availability of materials, indicating the persistence of the well-known mismatch between knowledge and practice in clinical practice.

Previous studies have shown that the nursing professionals’ level of knowledge regarding SPs was satisfactory^(22,23)^. Nonetheless, this practice is still not widely adopted by the majority^(22)^, especially given the low quality, availability, and accessibility of materials, which constitutes a significant obstacle to adherence^(24)^. Translating SP concepts into practice is therefore challenging, requiring support from the healthcare institution to promote adherence to standards across the entire team^(25)^.

The sociocognitive determinants related to “Organizational Constraint” presented the lowest average score on the global scale, indicating that nursing professionals did not consider unexpected situations (e.g., emergencies), limited time availability, higher workload, and complexity of SP measures to be obstacles to adherence. This result is concerning, given that these institutional limitations continue to be among the main practical obstacles to the consistent use of SPs, often overshadowing individual factors such as lack of knowledge or personal beliefs.

A study conducted in Saudi Arabia, aimed at investigating compliance with SPs among nurses and doctors, found that the main challenge reported that affected nurses’ adherence to SPs was excessive workload (82.1%)^(26)^. A scoping review conducted to identify shared factors related to nurses’ non-adherence to infection prevention and control practices established time as a particularly important resource in relation to adherence to SPs, mainly related to the time spent donning PPE. Furthermore, it highlighted that, in emergency situations, nurses reported that they frequently prioritize emergency care over infection prevention and control precautions^(24)^.

This discrepancy was identified in the quantitative data and the literature was also verified in the qualitative analysis, with participants indicating exceptions for not using SP, such as limited time availability and emergency situations. This incongruity can be partially explained by social desirability bias, common in self-report studies, where participants tend to respond according to socially expected behavior^(27,28)^. Moreover, it is possible that the stated intention, even under adverse conditions, may not materialize in daily practice, especially given the contextual factors mentioned, such as work overload, scarcity of resources, among others. Thus, it is possible to state that, although there is low identification of organizational restrictions as potential obstacles to adhering to SPs, the actual practice, best observed in the reports, is influenced by organizational and circumstantial factors, which highlights the need for institutional strategies that combine intention, training, and favorable structural conditions.

Analysis by professional categories indicated that, while nursing technicians perceived a greater “Social Influence” on adherence to SPs, nurses felt a greater impact from the “Organization” factor. This finding suggests different perceptions across hierarchical levels. For the technicians, the behavior of peers and superiors could be a motivating or discouraging factor, highlighting the importance of interpersonal relationships in promoting environments of positive engagement. For nurses, on the other hand, the institutional structure, which involves having materials in all workplaces, being trained, and having access to frequent training on SP seemed to be the main determinant of practice, which is consistent with the managerial role they often play.

A possible explanation for nursing technicians being more influenced by the work environment to adhere to SPs, especially when faced with the possibility of warnings for non-compliance, lies in the fact that, according to the law governing the nursing profession, these professionals’ activities can only be performed under the guidance and supervision of a nurse^(29)^, which can strengthen the role of authority figures in reinforcing adherence^(22)^.

In contrast, it is necessary to reflect that the imbalance in the perception of the possibility of warnings among nursing staff members may also indicate a culture of punitive surveillance, denoting an organizational environment more oriented towards sanctioning errors, which can compromise the genuine engagement of professionals in adopting precautionary practices. This finding is concerning because it may indicate that the current organizational culture in the study setting may not be actively promoting adherence to best practices in SPs^(25)^.

It is important to recognize that adherence is a personal decision, while conformity occurs predominantly through obedience to norms, often disregarding personal convictions. In this way, compliance with the SPs can be enhanced if factors influencing adherence are considered, especially those related to the behavior and attitudes of the nursing staff^(27)^. One aspect that needs to be considered in this context is the factor of “personal beliefs,” which, in this study, was not perceived by most participants as an obstacle to compliance with SPs, although the literature demonstrates that individual beliefs can directly impact personal risk perception and, consequently, the extent of protection adopted and risky behavior assumed^(13,19)^.

In this context, more than the possibility of warnings and punishments, the role of behavioral example should be encouraged, in which positive adherence practices exert a direct influence on other members of the multidisciplinary team. This result reinforces the importance of leading by example, with the potential to consolidate safer and more culturally positive environments^(14,30)^.

In what regards the “Organization” factor, training on SPs was highlighted as facilitating adherence, similar to findings in a study conducted in Brazil aimed at evaluating the socio-cognitive factors determining adherence to SPs by nursing professionals in clinical practice during the Covid-19 pandemic^(13)^. It has been identified that nursing professionals benefit from regular training sessions to improve their overall understanding of SPs and infection control, as well as the risks involved in non-adherence^(30)^.

Another mixed-methods study, conducted in Ethiopia to assess adherence to SPs for infection prevention practices and associated factors among healthcare professionals, showed that those who had received training in SPs were almost four times more likely to comply with them compared to those who had not^(28)^. However, it is emphasized that isolated training and awareness-raising actions are not sufficient to guarantee full adherence to the SPs. It is vital that healthcare organizations provide regular training and the necessary measures for all nurses, nursing technicians, and other healthcare professionals to enhance their competence and capabilities^(26)^.

Despite the stated intention to participate being high, qualitative data revealed contradictions, especially in contexts of urgency or overload. Nevertheless, intention is an important indicator of potential adherence, as it establishes awareness and a predisposition towards safe practices, which can be optimized through well-structured institutional interventions, exercising a mediating effect on performance related to infection control, being crucial for safe practices^(13)^.

The findings of this study have significant relevance to the practice of nursing professionals. By highlighting that organizational restrictions, including work overload, resource scarcity, and a punitive environment, outweigh individual factors, the study shifts the focus from individual accountability to the need for structural institutional interventions, reinforcing the role of organizational management, positive leadership, and a safety culture. The emphasis on behavioral example and leadership by example points to innovative strategies based on social modeling and transformational leadership. Thus, management can drastically influence the adherence behaviors of the nursing team by involving leaders as safety role models, encouraging open communication, acknowledging issues related to adherence to SPs, and supporting patient safety mechanisms in the workplace^(27)^.

This study has limitations inherent to the method adopted, which should be considered when interpreting the results. First, the cross-sectional nature prevents the assessment of causal relationships among the variables. Longitudinal studies would be required to analyze the persistence of these intentions and their realization over time. Another important limitation relates to the self-reporting of participants in the quantitative instrument (SPQ), which, as previously indicated, may be related to social desirability bias.

The study also presents a possible limitation related to the sample profile, composed exclusively of professionals from a single institution. This particularity limits the generalization of the results to other realities, since organizational conditions, access to materials, safety culture, and leadership style can vary considerably between different institutions and regions.

## CONCLUSION

The study showed that the nursing staff surveyed possessed satisfactory knowledge and a strong intention to adhere to standard precautions, recognizing their effectiveness in preventing infections. Nevertheless, significant challenges persisted related to organizational restrictions and the influence of interpersonal factors.

Furthermore, there is an urgent need for more collaborative environments, where positive leadership replaces punitive logic and the example of good practices is valued. While regular training is essential, it is not sufficient without adequate structural support. Therefore, continuous institutional interventions, integrating positive leadership and organizational improvements, are fundamental to strengthening effective adherence to SPs and consolidating a more solid and sustainable safety culture in healthcare practice.

## Data Availability

The entire dataset supporting the results of this study was published in the article itself.

## References

[1] Silva MCN, Machado MH (2020). Sistema de Saúde e Trabalho: desafios para a Enfermagem no
Brasil. Ciênc saúde coletiva.

[2] Drizaj E, Gugu M, Hidri S, Gaxhja E, Sula A, Tosuni S (2024). Occupational accidents with exposure to biological material in
nursing professionals – a cross-sectional study. Multidiscip Sci J.

[3] Bagheri M, Torabizadeh C, Doreh MA, Adelmanesh Y (2024). Assessment of operating room nurses’ exposure to biological
hazards: development and psychometric evaluation of a scale. BMC Nurs.

[4] Bouchoucha SL, Kilpatrick M, Lucas JJ, Phillips NM, Hutchinson A (2021). The Factors Influencing Adherence to Standard Precautions Scale –
Student version (FIASP- SV): a psychometric validation. Infect Dis Health.

[5] Brandão P, Luna TDC, Bazilio TR, Lam SC, Góes FGB, Pereira-Ávila FMV (2022). Compliance with standard precaution measures by health
professionals: comparison between two hospitals. Enferm Glob.

[6] Moshksar S, Nabavi MM, Danaei M, Momeni M, Askarian M (2023). Compliance with standard precautions, sharp injuries, and blood
and body fluid exposure among healthcare workers. Nurs Practice Today.

[7] Aboelfetoh EEE, Shakweer TT (2021). Effect of an Educational Guidelines on Compliance level regarding
Standard Precautions Measures among the operating room
nurses. Egypt J Health Care.

[8] Ahmed WM, Mohamed SS, Abu EI-fadl N (2020). Relationship between nurse’s knowledge and compliance with
Standard precautions in the operating room. J Nurs Sci Benha Univ.

[9] Porto JS, Marziale MHP (2020). Construction and validation of an educational video for improving
adherence of nursing professionals to standard precautions. Texto Contexto Enferm.

[10] Letvak S, Apple B, Jenkins M, Doss C, McCoy TP (2023). At risk safety behaviors of the perioperative nursing team: a
direct observational study. Healthcare (Basel).

[11] Dini G, Rahmani A, Montecucco A, Kusznir Vitturi B, Zacconi S, Manca A (2025). Occupational injuries and their determinants among healthcare
workers in western countries: a scoping review. Med Lav.

[12] Batran R, Ayed A, Batran A, Ejheisheh MA, Alassoud B, Hayek MF (2025). Determinants of nurses’ compliance with infection prevention and
control practices in critical care units. SAGE Open Nurs.

[13] Santos ASTD, Silva ACOE, Pereira-Ávila FMV, Coêlho HFC, Sousa LRM, Reis RK (2024). Sociocognitive factors determining compliance with standard
precautions by nursing professionals during the COVID-19
pandemic. Rev Bras Enferm.

[14] Huang Y, Lertwatthanawilat W, Klunklin P, Unahalekhaka A (2024). A causal model of factors influencing adherence to standard
precautions practices among chinese emergency nurses: a cross-sectional
study. PRIJNR.

[15] Cunha QBD, Freitas EO, Pai DD, Santos JLGD, Silva RMD, Camponogara S (2024). Adherence to standard precautions in university hospitals during
the COVID-19 pandemic: a mixed study. Rev Esc Enferm USP.

[16] Creswell JW, Clark VLP (2013). Projeto de pesquisa: métodos qualitativo, quantitativo e misto.

[17] Michinov E, Buffet-Bataillon S, Chudy C, Constant A, Merle V, Astagneau P (2016). Sociocognitive determinants of self-reported compliance with
standard precautions: development and preliminary testing of a questionnaire
with French health care workers. Am J Infect Control.

[18] Luna TDC, Pereira-Ávila FMV, Brandão P, Michinov E, Góes FGB, Caldeira NMVP (2020). Psychometric properties of the Brazilian version of the Standard
Precautions Questionnaire for health professionals in Brazil. Rev Bras Enferm.

[19] Pereira-Ávila FMV, Michinov E, Luna TDC, Conde PS, Pereira-Caldeira NMV, Góes FGB (2019). Standard precautions questionnaire: cultural adaptation and
semantic validation for health professionals in Brazil. Cogitare Enferm.

[20] Chicco D, Sichenze A, Jurman G (2025). A simple guide to the use of Student’s t-test, Mann-Whitney U
test, Chi-squared test, and Kruskal-Wallis test in
biostatistics. BioData Min.

[21] Bardin L (2015). Tradução de Luís Antero Reto e Augusto Pinheiro.

[22] Ghabayen F, ALBashtawy M, Abdelkader RH, Jarrah S, Eshah N, Abdalrahim A (2023). Knowledge and compliance with standard precautions among
nurses. SAGE Open Nurs.

[23] Al-Faouri I, Okour SH, Alakour NA, Alrabadi N (2021). Knowledge and compliance with standard precautions among
registered nurses: a cross-sectional study. Ann Med Surg (Lond).

[24] McCauley L, Kirwan M, Matthews A (2021). The factors contributing to missed care and non-compliance in
infection prevention and control practices of nurses: a scoping
review. Int J Nurs Stud Adv.

[25] van Gulik N, Bouchoucha S, Apivanich S, Lucas J, Hutchinson A (2021). Factors influencing self-reported adherence to standard
precautions among Thai nursing students: a cross sectional
study. Nurse Educ Pract.

[26] Elseesy NAM, Al-Zahrani AE, Kandil FS, Mahsoon A, Elhady MM (2023). Compliance among registered nurses and doctors in critical care
units: challenges affecting their adherence to standard
precautions. Healthcare (Basel).

[27] Berdida DJE, Grande RAN (2024). Nurses’ safety climate, quality of care, and standard precautions
adherence and compliance: a cross-sectional study. J Nurs Scholarsh.

[28] Kassa A, Tadesse SE, Walelign F, Kebede N (2022). Compliance with standard precaution of infection prevention
practice and associated factors among health care workers in Ethiopia: mixed
method study. Health Sci Rep.

[29] Brasil (1986). Dispõe sobre a regulamentação do exercício da enfermagem, e dá
outras providências. Lei n° 7.498, de 25 de junho de 1986.

[30] Lim SH, Bouchoucha SL, Aloweni F, Bte Suhari N’ (2021). Evaluation of infection prevention and control preparedness in
acute care nurses: factors influencing adherence to standard
precautions. Infect Dis Health.

