# Supplementary material for: Sociocognitive determinants of adherence to standard precautions by the nursing staff in a regional hospital: a mixed-methods study
Source: Rev Esc Enferm USP. 2026 Mar 23;60:e20250335. doi: 10.1590/1980-220X-REEUSP-2025-0335en (PMC13041239; doi:10.1590/1980-220X-REEUSP-2025-0335en)
Supplement: Translation

# Translation: Determinantes sociocognitivos na adesão às precauções-padrão pela equipe de enfermagem em hospital regional: estudo misto

**DOI:** 10.1590/1980-220X-REEUSP-2025-0335pt

**Authors:** Junaiza Rodrigues Lima Nunes, Cleydson Wendel Nunes de Souza, Allyne Brena Coimbra Azevedo, Cristianne Teixeira Carneiro, Mychelangela de Assis Brito, Maria Augusta Rocha Bezerra, Ruth Cardoso Rocha

**Affiliations:** 1 Universidade Federal do Piauí, Floriano, PI, Brasil.

**Keywords:** Saúde do trabalhador, Precauções Universais, Enfermagem

## Abstract

**Objetivo:**

Analisar os determinantes sociocognitivos na adesão às precauções-padrão pela equipe de enfermagem em hospital regional brasileiro.

**Método:**

Estudo de método misto, design sequencial explanatório, realizado entre janeiro de 2023 e março de 2024, com 230 profissionais. Utilizaram-se da versão brasileira do *Standard Precautions Questionnaire* e das entrevistas semiestruturadas. A análise quantitativa empregou o teste de Mann-Whitney e a qualitativa, análise de conteúdo.

**Resultados:**

A média geral de adesão sociocognitiva foi de 3,90 (DP = 0,48), com maior escore no fator “Atitude” (4,60; DP = 0,48) e menor em “Restrições organizacionais” (2,95; DP = 1,17). Identificaram-se diferenças significativas entre categorias profissionais em “Influência Social” (enfermeiros: 4,72; técnicos: 4,79; p = 0,001) e “Organização” (enfermeiros: 4,60; técnicos: 4,49; p = 0,033). A integração dos achados demonstrou convergência entre dados quantitativos e qualitativos, evidenciando que a adesão, embora satisfatória, é influenciada por fatores laborais.

**Conclusões:**

Técnicos demonstraram maior influência social, enquanto enfermeiros identificaram mais barreiras organizacionais. O exemplo entre pares favorece a adesão, apesar do persistente receio de advertências, diante do não cumprimento das práticas.

## INTRODUÇÃO

Os profissionais de enfermagem representam parcela significativa da força de trabalho em ambientes hospitalares^(1)^, sendo expostos a diferentes riscos ocupacionais de natureza biológica, química, física e ergonômica^(2)^. Nesse contexto, os riscos biológicos representam uma das ameaças mais comumente enfrentadas^(3)^, envolvendo contato com sangue, tecidos ou outros fluidos corporais potencialmente infecciosos^(2)^.

Para mitigá-los e prevenir a ocorrência de lesões, é imprescindível o acesso e a adesão às medidas de biossegurança, especialmente às Precauções-Padrão (PP), que visam limitar a transmissão de agentes infecciosos nos ambientes de assistência à saúde^(4)^, como a higienização das mãos, o uso de Equipamentos de Proteção Individual (EPI) – como luvas, aventais, óculos e máscaras – e o manuseio seguro de materiais perfurocortantes e contaminados^(5)^.

Embora os profissionais de enfermagem apresentem maior adesão às PP, em comparação com outros profissionais da saúde^(6)^, os índices permanecem aquém do ideal^(7)^. Observa-se que até 68,3% dos enfermeiros possuem conhecimento insatisfatório sobre as PP e 73,3% não as adotam sistematicamente^(8)^. As razões frequentemente associadas a essa não adesão incluem desorganização laboral, sobrecarga de trabalho, jornadas duplas, situações de urgência, escassez de materiais e fatores individuais, como esquecimento e baixa qualificação profissional^(9)^.

Esse contexto tem contribuído para o aumento da incidência de lesões ocupacionais entre profissionais de enfermagem^(10)^, cuja prevalência, ao longo da carreira, tem atingido mais da metade dos enfermeiros^(11)^. Desta forma, torna-se indispensável identificar os motivos subjacentes à não adesão às PP^(4)^, visando suprir lacunas identificadas em estudos anteriores^(12,13)^ e aprofundar a compreensão dos determinantes que influenciam essa prática.

Entre os fatores que influenciam o comportamento humano e a tomada de decisão dos profissionais de enfermagem, destacam-se os determinantes sociocognitivos, como o conhecimento, a motivação, a intenção, as expectativas e as percepções^(13)^. Esses elementos desempenham papel fundamental na forma como esses profissionais aderem às práticas de PP, especialmente em contexto caracterizado por alta complexidade, dinamismo e situações frequentemente urgentes^(14)^.

No entanto, observa-se lacuna na literatura científica no que se refere a estudos que abordem especificamente os determinantes sociocognitivos e a relação com o comportamento dos profissionais de enfermagem nesse cenário. Essa constatação reforça a necessidade de maior compreensão, reconhecimento e avaliação crítica desses fatores^(13)^. A incorporação de dados mistos na análise contribui para interpretação mais aprofundada, permitindo analisar com mais abrangência esse fenômeno^(15)^. Diante do exposto, objetivou-se analisar os determinantes sociocognitivos que influenciam a adesão às precauções-padrão por parte da equipe de enfermagem em hospital regional brasileiro.

## MÉTODO

### Tipo de Estudo

O estudo utilizou método misto, com desenho sequencial explanatório (QUAN→qual), no qual a fase quantitativa antecedeu e orientou a análise qualitativa. Os achados qualitativos foram empregados para aprofundar a interpretação dos resultados quantitativos^(16)^. A etapa quantitativa foi transversal e a qualitativa apresentou caráter analítico. Para garantir rigor metodológico, aplicou-se o *Mixed Methods Appraisal Tool* (MMAT). A investigação buscou responder à questão: quais determinantes sociocognitivos influenciam a adesão às Precauções-Padrão pela equipe de enfermagem em hospital regional brasileiro?

### Local

A pesquisa foi realizada em hospital regional, localizado no interior do estado do Piauí, Brasil. Trata-se de hospital geral, com serviços cirúrgicos, clínicos, obstétricos, pediátricos, entre outros, cuja principal função é a assistência à saúde. Além disso, a instituição desenvolve atividades secundárias, como apoio diagnóstico, atendimento a emergências, dispensação de medicamentos, imunização, promoção da saúde, prevenção de doenças e prestação de cuidados de complexidade média e alta. Em 2023, a instituição contava com 745 leitos, divididos nos seguintes setores: Centro Cirúrgico, Centro de Material e Esterilização, Clínica Cirúrgica, Clínica Médica, Obstetrícia, Pediatria, Pronto Socorro e Unidade de Terapia Intensiva.

### População e Critérios de Seleção

A população do estudo foi composta por 377 profissionais de enfermagem atuantes no hospital, sendo 112 enfermeiros e 265 técnicos de enfermagem, conforme dados fornecidos pela instituição. Estabeleceu-se como critérios de inclusão: ser profissional da equipe de enfermagem atuante no hospital regional há, no mínimo, um ano, e ter contato direto com o paciente. Excluíram-se aqueles afastados por férias ou licença médica.

### Definição da Amostra

A amostra foi selecionada por conveniência, e os participantes foram recrutados presencialmente e de forma individual, nos diferentes setores da instituição. Para a coleta quantitativa, realizou-se cálculo amostral, com objetivo de assegurar número significativo de participantes, adotando nível de confiança de 95% e margem de erro de 5%, o que resultou em amostra mínima estimada de 191 profissionais de enfermagem. Contudo, a amostra final foi ampliada, totalizando 230 participantes na fase quantitativa.

Para coleta qualitativa, a seleção dos participantes ocorreu por amostragem intencional (*purposeful sampling*), considerando critérios de relevância para compreensão aprofundada do fenômeno investigado. Buscou-se diversidade de experiências profissionais, cargos e setores de atuação, de modo a garantir proporcionalidade entre enfermeiros e técnicos de enfermagem e contemplar diferentes perspectivas sobre as PP. O encerramento amostral aconteceu por saturação teórica, alcançada quando novas entrevistas deixaram de acrescentar informações substancialmente novas às categorias analíticas, resultando no total de 15 participantes nessa fase.

### Coleta de Dados

A coleta de dados foi realizada entre janeiro de 2023 e março de 2024. Na fase quantitativa, foram coletados dados de caracterização sociodemográfica (sexo; idade; etnia; renda) e formação profissional (categoria de formação; tempo de formação; setor de atuação; turno de trabalho; quantidade de empregos; satisfação com o emprego; tempo de trabalho na profissão; tempo de trabalho na instituição; e doença relacionada ao trabalho).

Em seguida, utilizou-se da versão brasileira do *Standard Precautions Questionnaire* (SPQ), originalmente desenvolvido e validado na França, com objetivo de avaliar os determinantes sociocognitivos na adesão às PP, como atitudes, comportamentos, limitações pessoais e organizacionais^(17)^. O SPQ passou por adaptação cultural e validação semântica para o português brasileiro, apresentando propriedades psicométricas satisfatórias, incluindo validade de construto e confiabilidade^(18)^. O instrumento é composto por 24 itens distribuídos em sete fatores: 1 – Comportamento interpessoal (2 itens), 2 – Restrições organizacionais (4 itens), 3 – Intenção de seguir as PP (4 itens), 4 – Influência social (4 itens), 5 – Atitudes em relação às PP (3 itens), 6 – Organização (3 itens), e 7 – Restrições individuais (4 itens)^(18,19)^.

A fase qualitativa foi conduzida com base em roteiro semiestruturado, elaborado para explorar a percepção dos profissionais de enfermagem sobre a aplicação das PP. As entrevistas, com duração média de 10 minutos, foram realizadas individualmente, em sala reservada, gravadas em áudio e transcritas integralmente. Antes do início dessa etapa, o roteiro foi validado com enfermeiros de outro serviço, que não foram incluídos na amostra final, o que permitiu ajustes no instrumento e aperfeiçoamento da técnica de entrevista. Enfatiza-se, ainda, que os três pesquisadores assistentes, responsáveis pela coleta de dados, realizaram treinamento prévio, orientado pela pesquisadora principal.

### Análise e Tratamento dos Dados

Os dados quantitativos foram organizados no Microsoft Excel® 2016, por meio de dupla digitação e, posteriormente, analisados no software SPSS®, versão 20.0. Utilizou-se da estatística descritiva, incluindo medidas de tendência central e dispersão, bem como o teste de Shapiro-Wilk para verificação da aderência das variáveis à distribuição normal. Ao considerar a ausência de normalidade para parte dos dados, aplicou-se o teste não paramétrico de Mann-Whitney para comparar os escores do instrumento entre as categorias profissionais^(20)^. Adotaram-se nível de significância de p ≤ 0,05 e intervalo de confiança de 95%.

Os dados qualitativos foram organizados em três etapas: pré-análise, em que o material foi organizado para torná-lo funcional e sistematizar as ideias iniciais; levantamento/categorização do material, na qual o material foi examinado, foram definidas as categorias e unidades de registro e contexto. Por último, na interpretação dos resultados, procedeu-se à descrição analítica, em que o material foi submetido a estudo aprofundado, guiado por hipóteses e referenciais teóricos^(21)^. A integração dos dados foi realizada mediante a conexão entre os resultados quantitativos e qualitativos, buscando convergências e divergências e aprofundamento da análise do objeto de estudo.

### Aspectos Éticos

Este estudo respeitou as diretrizes do Conselho Nacional de Saúde para pesquisas com seres humanos, conforme as Resoluções nº 466/2012, 510/2016 e 580/2018, tendo sido aprovado pelo Comitê de Ética em Pesquisa da Universidade Federal do Piauí, sob pareceres nº 5.834.174 e nº 6.086.132. A identidade dos participantes foi preservada por meio da atribuição de códigos alfanuméricos (P1, P2, P3…), e todos assinaram o Termo de Consentimento Livre e Esclarecido (TCLE).

## RESULTADOS

Participaram da pesquisa 230 profissionais de enfermagem do hospital regional, com idades entre 19 e 70 anos. A maioria era do sexo feminino (81,7%), com predominância de jovens entre 19 e 40 anos (80,0%). Em relação à etnia, 54,4% autodeclararam-se negros. Predominaram técnicos de enfermagem (66,1%), com tempo de formação entre seis e dez anos (30,4%) e atuação nos três turnos (56,1%). A maioria possuía apenas um vínculo empregatício (68,7%) e salário entre R$ 1.000,00 e R$ 3.000,00 (76,5%), além de elevada satisfação profissional (86,5%). Quanto ao tempo de trabalho, prevaleceu o intervalo de um a cinco anos tanto na profissão (43,5%) quanto na instituição (47,0%). Por fim, 75,3% relataram não apresentar doenças ocupacionais. Os dados descritos estão detalhados na Tabela 1.

**Tabela 1:** Caracterização socioeconômica e profissional dos profissionais de enfermagem atuantes em hospital regional – PI, Brasil, 2023–2024.

Variáveis	N	%	Média ± Dp
Sexo			
Feminino	188	81,7	
Masculino	42	18,3	
Idade			33,88 ± 9,75
19–40	184	80,0	
41–70	46	20,0	
Etnia			
Negro	125	54,4	
Pardo	76	33,0	
Branco	28	12,2	
Indígena	1	0,4	
Categoria de formação			
Técnico em Enfermagem	152	66,1	
Enfermeiro	78	33,9	
Tempo de formação (anos)			
1–5	95	41,3	
6–10	70	30,4	
> 10	64	27,9	
Não informado	1	0,4	
Setor de atuação			
Centro Cirúrgico	51	22,2	
Obstetrícia	37	16,1	
Pronto Socorro	31	13,5	
Clínica Médica	29	12,6	
Unidade de Terapia Intensiva	27	11,7	
Clínica Cirúrgica	22	9,6	
Centro de Material e Esterilização	21	9,1	
Pediatria	12	5,2	
Turno de trabalho			
Matutino/Vespertino/Noturno	129	56,1	
Matutino	33	14,3	
Matutino/Noturno	22	9,6	
Matutino/Vespertino	15	6,5	
Noturno	14	6,2	
Vespertino	10	4,3	
Vespertino/Noturno	6	2,6	
Não informado	1	0,4	
Quantidade de empregos			
Um	158	68,7	
Dois	61	26,5	
Três ou mais	9	3,9	
Não informado	2	0,9	
Renda (salários-mínimos)			
1–3	176	76,5	
3–6	48	20,9	
> 6	5	2,2	
Não informado	1	0,4	
Satisfação com o emprego			
Sim	199	86,5	
Não	31	13,5	
Tempo de trabalho na profissão (anos)	
< 1	46	20,0	
1–5	100	43,5	
6–10	38	16,5	
> 10	45	19,6	
Não informado	1	0,4	
Tempo de trabalho na instituição (anos)			
< 1	58	25,2	
1–5	108	47,0	
6–10	36	15,7	
> 10	28	12,1	
Doença relacionada ao trabalho			
Sim	47	20,4	
Não	173	75,3	
Não sei informar	10	4,3	

Na Tabela 2, apresentam-se os resultados obtidos com a aplicação do questionário *Standard Precautions Questionnaire* (SPQ). No fator “Atitude” (parte 1), majoritariamente, os participantes concordaram totalmente que as PP constituem forma eficaz para prevenir as infecções hospitalares (68,3%), bem como para proteger os pacientes (67,4%) e profissionais (67,4%). No fator “Influência Social”, 36,1% acreditaram parcialmente que colegas valorizavam as PP. Contudo, 48,7% referiram risco de advertência por superiores quando não as seguem, sendo o percentual mais prevalente neste domínio. Quanto ao fator “Organização”, destacaram-se três itens com prevalência acima de 60%: 65,7% relataram total eficácia da disponibilidade de material; 60,4% indicaram que estar capacitado favorece a adesão; e 61,3% reforçaram a importância de capacitações frequentes.

**Tabela 2:** Distribuição, percentual, média e desvio padrão, segundo itens da versão brasileira do *Standard Precautions Questionnaire*, respondidos pelos profissionais de enfermagem – PI, Brasil, 2023–2024.

Itens	Nada eficaz n (%)	Um pouco eficaz n (%)	Mais ou menos eficaz n (%)	Parcialmente eficaz n (%)	Totalmente eficaz n (%)	Não respondeu n (%)	Média	Desvio Padrão
**Parte 1– Atitude**								
As precauções-padrão são medidas eficazes para reduzir as infecções hospitalares	5 2,2%	–	8 3,5%	59 25,7%	157 68,3%	1 0,4%	4,59	0,76
Se eu seguir as medidas de precauções-padrão, protegerei meus pacientes de uma infecção	–	–	9 3,9%	65 28,3%	155 67,4%	1 0,4%	4,64	0,56
Seguir as medidas de precauções-padrão vai me proteger de uma infecção	1 0,4%	2 0,9%	11 4,8%	62 27%	154 67,0%	–	4,59	0,66
**Parte 2 – Influência social**								
A maioria dos meus colegas de trabalho pensa que é importante seguir as precauções-padrão	4 1,7%	14 6,1%	70 30,4%	83 36,1%	57 24,8%	2 0,9%	3,78	0,97
Corro o risco de receber advertências dos meus superiores se não seguir as precauções-padrão	5 2,2%	8 3,5%	22 9,6%	83 36,1%	112 48,7%	–	4,25	0,92
Corro o risco de receber advertências dos enfermeiros e auxiliares responsáveis pela higienização se não seguir as precauções-padrão	8 3,5%	16 7,0%	27 11,7%	87 37,8%	90 39,1%	1 0,4%	4,03	1,06
Corro o risco de receber advertências dos médicos se não seguir as precauções-padrão	19 8,3%	19 8,3%	38 16,5%	69 30,0%	83 36,1%	2 0,9%	3,80	1,26
**Parte 3 – Organização**								
Ter material (qualidade, disponibilidade e acessibilidade) em todos os locais de trabalho	1 0,4%	4 1,7%	19 8,3%	54 23,5%	151 65,7%	1 0,4%	4,53	0,76
Estar capacitado no que se refere às precauções-padrão	1 0,4%	2 0,9%	16 7,0%	70 30,4%	139 60,4%	2 0,9%	4,52	0,71
Ter capacitações quanto às precauções-padrão	2 0,9%	2 0,9%	10 4,3%	74 32,2%	141 61,3%	1 0,4%	4,53	0,70
**Parte 4 – Comportamento interpessoal**								
Quando o profissional médico tem um comportamento exemplar em relação às precauções-padrão	3 1,3%	14 6,1%	28 12,2%	68 29,6%	116 50,4%	1 0,4%	4,23	0,97
Quando os meus colegas de trabalho têm um comportamento exemplar em relação às precauções-padrão	8 3,5%	7 3,0%	30 13,0%	76 33,0%	108 47,0%	1 0,4%	4,18	1,01
**Itens**	**Não é obstáculo n (%)**	**Um pouco de obstáculo n (%)**	**Mais ou menos obstáculo n (%)**	**Parcialmente obstáculo n (%)**	**É um obstáculo n (%)**	**Não respondeu n (%)**	**Média**	**Desvio Padrão**
**Parte 5 – Restrições organizacionais**								
Situações inesperadas que podem atrapalhar a realização de meu trabalho (urgência, solicitação de colegas, nova tarefa a cumprir).	44 19,1%	16 7,0%	60 26,1%	52 22,6%	56 24,3%	2 0,9%	3,28	1,42
Falta de tempo.	89 38,7%	32 13,9%	36 15,7%	30 13,0%	41 17,8%	2 0,9%	2,60	1,56
Carga de trabalho mais elevada que de costume.	56 24,3%	31 13,5%	52 22,6%	30 13,0%	58 25,2%	3 1,3%	3,05	1,54
Complexidade das medidas de precauções-padrão.	66 28,7%	32 13,9%	50 21,7%	33 14,3%	46 20,0%	3 1,3%	2,87	1,53
**Parte 6 – Restrições individuais**								
Falta de conhecimento sobre as precauções-padrão.	55 23,9%	21 9,1%	33 14,3%	27 11,7%	91 39,6%	3 1,3%	3,37	1,65
Rotina, hábitos e equipe de trabalho.	81 35,2%	22 9,6%	40 17,4%	28 12,2%	57 24,8%	2 0,9%	2,84	1,63
Crenças pessoais relacionadas às precauções-padrão.	88 38,3%	21 9,1%	42 18,3%	24 10,4%	52 22,6%	3 1,3%	2,73	1,64
Problemas relacionados ao material (qualidade, disponibilidade e acessibilidade).	35 15,2%	15 6,5%	37 16,1%	44 19,1%	97 42,2%	2 0,9%	3,69	1,47
**Itens**	**Nunca n (%)**	**Raramente n (%)**	**Às vezes n (%)**	**Muitas vezes n (%)**	**Sempre n (%)**	**Não respondeu n (%)**	**Média**	**Desvio Padrão**
**Parte 7 – Intenção de seguir as precauções-padrão**								
Mesmo quando o paciente for difícil.	3 1,3%	6 2,6%	24 10,4%	29 12,6%	167 72,6%	1 0,4%	4,53	0,88
Mesmo quando houver pouco tempo.	2 0,9%	11 4,8%	25 10,9%	39 17,0%	152 66,1%	1 0,4%	4,43	0,93
Mesmo quando minhas mãos estiverem doloridas ou machucadas.	18 7,8%	7 3,0%	22 9,6%	44 19,1%	139 60,4%	–	4,21	1,21
Mesmo em situação de urgência	5 2,2%	10 4,3%	21 9,1%	26 11,3%	167 72,6%	1 0,4%	4,49	0,98

No “Comportamento Interpessoal”, 50,4% apontaram que o comportamento exemplar de médicos facilita a adesão e 47,0% reforçaram a influência positiva dos colegas. Sobre “Restrições Organizacionais”, o principal obstáculo foi “situações inesperadas” (24,3%), ao passo que “falta de tempo” não foi considerada obstáculo por 38,7%. Em “Restrições Individuais”, o maior obstáculo foi material inadequado (42,2%), enquanto crenças pessoais foram minimizadas (38,3% declararam não serem obstáculos). Por fim, em “Intenção”, a maioria sempre seguia as PP, especialmente em situações difíceis (72,6%).

A Escala SPQ apresentou média geral de 3,90 (DP= 0,48). O fator “Atitude” obteve maior pontuação (4,60; DP: 0,48), enquanto o fator “Restrições organizacionais” registrou a menor média (2,95; DP: 1,17). Na comparação por categoria profissional (Tabela 3), os técnicos de enfermagem apresentaram escore significativamente maior em “Influência social” (4,09) em relação aos enfermeiros (3,72; p = 0,001), enquanto os enfermeiros pontuaram mais alto no fator “Organização” (4,60), comparados aos técnicos (4,49; p = 0,033). Para os fatores “Atitude”, “Comportamento interpessoal”, “Restrições organizacionais”, “Restrições individuais” e “Intenção de seguir as precauções padrão”, não foram observadas diferenças estatísticas relevantes entre os grupos.

**Tabela 3:** Escore de determinantes sociocognitivos referente aos fatores da versão brasileira do *Standard Precautions Questionnaire* por categoria profissional dos profissionais de enfermagem – PI, Brasil, 2023–2024.

Fatores	Categorias profissionais		Valor de p*
	Enfermeiro	Técnico de Enfermagem	
Atitude	4,64	4,58	0,293
Influência social	3,72	4,09	**0,001**
Organização	4,60	4,49	**0,033**
Comportamento interpessoal	4,07	4,27	0,331
Restrições organizacionais	3,06	2,89	0,250
Restrições individuais	3,14	3,17	0,947
Intenção de seguir as precauções padrão	4,42	4,41	0,870

*Teste de Mann-Whitney.

No tocante à análise qualitativa do estudo, o conteúdo das entrevistas possibilitou a constituição de três categorias analíticas: Compreensão dos profissionais de enfermagem sobre as precauções-padrão, Utilização das precauções-padrão pela equipe de enfermagem e Fatores que dificultam o uso das precauções-padrão pela equipe de enfermagem. Essas categorias foram incorporadas aos resultados quantitativos, com a extração de declarações significativas para melhor representá-los, conforme descrito no *Joint display*, exibido no Quadro 1.

**Quadro 1:** *Joint display* com integração dos resultados quantitativos e declarações dos participantes – PI, Brasil, 2023–2024.

Subcategoria	Resultados quantitativos (n = 230)	Resultados qualitativos (n = 15)
**Categoria 1 – Compreensão dos profissionais de enfermagem sobre as precauções-padrão**		
Entendimento quanto ao conceito das PP.	68,3% indicaram que as PP são medidas eficazes para reduzir as infecções hospitalares. 39,6% indicaram que a falta de conhecimento sobre as PP é um obstáculo. 38,3% indicaram que crenças pessoais relacionadas às PP não são obstáculo para adesão. 60,4% indicaram que estar capacitado no que se refere às PP é totalmente eficaz na adesão. 61,3% indicaram que ter capacitações quanto às PP é totalmente eficaz na adesão.	*A precaução vem de se precaver, de profilaxia, prevenção, e padrão é porque é uma forma já pronta a ser seguida, então, o objetivo da precaução-padrão é buscar padronizar formas a serem seguidas por todos os profissionais de saúde, com o objetivo de prevenir ou até mesmo de controlar a transmissão de micro-organismos no ambiente de trabalho, principalmente na questão na área hospitalar*. (P13)
Indicação sobre as principais medidas de PP utilizadas.	65,7% indicaram que ter material (qualidade, disponibilidade e acessibilidade) em todos os locais de trabalho é eficaz. 42,2% indicaram que problemas relacionados ao material (qualidade, disponibilidade e acessibilidade) são obstáculos à adesão às PP.	*Equipamentos de proteção individual, óculos, máscaras, lavagem correta das mãos, higienização do ambiente crítico, por exemplo, como o do centro cirúrgico, de equipamentos, colocar os materiais perfurocortantes no local certo e tudo isso evita possíveis acidentes de trabalho e/ou alguma contaminação*. (P1)
Percepção de que o uso das PP previne acidentes e protege o profissional e o paciente contra doenças.	67,4% indicaram que se seguir as medidas de PP, protegerá os pacientes de uma infecção. 67,0% indicaram que seguir as medidas de PP protegerá o profissional de uma infecção.	*Elas [PP] previnem problemas ou algum tipo de infecção tanto do paciente para o profissional quanto do profissional para o paciente*. (P1)
**Categoria 2 – Utilização das precauções-padrão pela equipe de enfermagem**		
Quando devem ser utilizadas as PP.	72,6% indicaram que sempre têm intenção de seguir as PP, mesmo quando o paciente for difícil. 66,1% indicaram que sempre têm intenção de seguir as PP, mesmo quando houver pouco tempo. 60,4% indicaram que sempre têm intenção de seguir as PP, mesmo quando as mãos estiverem doloridas ou machucadas. 72,6% indicaram que sempre têm intenção de seguir as PP, mesmo em situação de urgência. 38,7% indicaram que falta de tempo não é um obstáculo para adesão às PP.	*[…] ela deve ser usada sempre na assistência de um paciente e outro […] vai haver exceções? Vai! Dependendo do caso, mas é recomendado que elas sejam usadas sempre*. (P13) *[…] em caso de emergência, digamos que tenha uma parada cardiorrespiratória: você está fazendo um procedimento simples qualquer e necessita reanimar e não dá tempo para se paramentar por completo, mas, aí, a gente usa o que já está ali por perto*. (P12)
**Categoria 3 – Fatores que interferem no uso das precauções-padrão pela equipe de enfermagem**		
Negligência, esquecimento, falta de cuidado, descuido e alergia.	42,2% indicaram que problemas relacionados ao material (qualidade, disponibilidade e acessibilidade) são obstáculos para a adesão às PP. 25,2% indicaram que carga de trabalho mais elevada que de costume é um obstáculo para a adesão às PP.	*De qualquer maneira, é negligência: você tem que se policiar o tempo todo, para estar sempre dentro dos padrões*. (P7) *Mesmo sabendo da importância, às vezes, não uso porque não tem tempo; às vezes, relaxa […] a gente vai confiando, mesmo sabendo que “quem vê cara, não vê doença”; e, às vezes, nem uso tanto, porque eu sou alérgica ao látex*. (P5) *Quando não há EPI disponibilizado pela instituição, quando há uma falta ou que não foi reabastecido […]*. (P13) *Você deseja que eu seja sincero? Bom, sendo bastante sincero, quando eu não uso as precauções-padrão é por falta de cuidado, unicamente por isso, mesmo sabendo que elas protegem os profissionais e pacientes*. (P15)
Indisponibilidade de materiais.	65,7% indicaram que ter material (qualidade, disponibilidade e acessibilidade), em todos os locais de trabalho, é totalmente eficaz na adesão às PP.	*[…] deixar de usar porque a gente quer, a gente não deixa, só deixa de usar mesmo quando não tem. E é difícil faltar, mas, às vezes, faltam e quando falta, a gente improvisa*. (P1)
Influência coletiva.	36,1% indicaram que é parcialmente eficaz, quando a maioria dos colegas de trabalho pensa que é importante seguir às PP. 50,4% indicaram que é totalmente eficaz, quando o profissional médico tem um comportamento exemplar em relação às PP. 47,0% indicaram que é totalmente eficaz, quando os colegas de trabalho têm um comportamento exemplar em relação às PP.	*[…] porque quando eu estou usando o meu EPI, eu estou segura: não vou nem pegar e nem muito menos transmitir. Se todo mundo usasse as medidas de precaução, seria mais interessante, mas nem todo mundo usa*. (P9)

**Nota:** PP = precauções-padrão.

A integração dos resultados evidencia forte convergência entre as dimensões avaliadas, sobretudo no reconhecimento da importância das PP e na percepção da eficácia para prevenir infecções, aspecto corroborado tanto pelos elevados escores de “Atitude”, no componente quantitativo, quanto pelos relatos que destacaram o caráter preventivo e a proteção mútua proporcionada pelas medidas. Também, observou-se convergência na identificação de barreiras organizacionais, especialmente no que se refere à disponibilidade e adequação de materiais, apontada quantitativamente como principal obstáculo e reiterada qualitativamente pela menção à falta de EPI e necessidade de improvisação. A influência coletiva igualmente se mostrou consistente entre métodos: embora a análise quantitativa tenha indicado moderada influência social, os discursos reforçaram que o comportamento exemplar de colegas e médicos favorece a adesão.

Por outro lado, emergiram divergências sutis quanto à utilização efetiva das PP: enquanto os dados quantitativos sugeriram elevada intenção de adesão, mesmo em situações adversas, os depoimentos qualitativos revelaram situações de negligência, esquecimento e improviso, indicando descompasso entre intenção declarada e prática cotidiana. De modo geral, a convergência dos achados sustenta que o conhecimento, a disponibilidade de recursos e o ambiente coletivo constituíram determinantes centrais da adesão, enquanto as divergências destacaram a necessidade de estratégias institucionais voltadas à redução de barreiras práticas e comportamentais.

## DISCUSSÃO

A análise dos determinantes sociocognitivos da equipe de enfermagem de um hospital regional brasileiro evidenciou avanços importantes no conhecimento e na intenção declarada de enfermeiros e técnicos de enfermagem quanto à adesão às PP, especialmente quanto ao reconhecimento da eficácia na prevenção de infecções hospitalares. Entretanto, essa percepção positiva contrasta com as barreiras práticas identificadas, particularmente no que diz respeito às restrições organizacionais e à disponibilidade de materiais, indicando a persistência do conhecido descompasso entre o saber e o fazer na prática clínica.

Estudos anteriores demonstraram que o nível de conhecimento dos profissionais de enfermagem sobre as PP era satisfatório^(22,23)^. Contudo, essa prática ainda é pouco incorporada pela maioria^(22)^, especialmente diante da baixa qualidade, disponibilidade e acessibilidade de materiais, o que configura importante obstáculo à adesão^(24)^. A tradução dos conceitos de PP em prática é, portanto, desafiadora, sendo necessário o apoio da instituição de saúde para promover a adesão aos padrões por toda a equipe^(25)^.

Os determinantes sociocognitivos relativos à “Restrição organizacional” apresentaram a menor pontuação média na escala global, indicando que os profissionais de enfermagem não consideraram que situações inesperadas (p.ex., urgências), pouca disponibilidade de tempo, carga de trabalho mais elevada e complexidade das medidas de PP constituem obstáculos para a adesão. Esse resultado é preocupante, visto que essas limitações institucionais continuam configurando entre os maiores entraves práticos para o uso consistente das PP, sobrepondo-se, muitas vezes, a fatores individuais, como desconhecimento ou crenças pessoais.

Estudo realizado na Arábia Saudita, com objetivo de investigar a conformidade com as PP entre enfermeiros e médicos, identificou que o principal desafio relatado que afetou a adesão dos enfermeiros às PP foi excesso de carga de trabalho (82,1%)^(26)^. Revisão de escopo realizada com objetivo de identificar os fatores compartilhados relacionados a não adesão dos enfermeiros às práticas de prevenção e ao controle de infecções estabeleceu o tempo como recurso de particular importância em relação à adesão às PP, relacionado principalmente ao tempo gasto para vestir o EPI. Ademais, destacou que, em situações de urgência, enfermeiros relataram que frequentemente priorizam o atendimento de emergência acima das precauções de prevenção e controle de infecções^(24)^.

Essa divergência foi identificada nos dados da etapa quantitativa e a literatura também foi verificada na análise qualitativa, com os participantes indicando exceções para não usar a PP, como pouca disponibilidade tempo e situações de urgência. Essa incongruência pode ser parcialmente explicada pelo viés de desejabilidade social, comum em estudos de autorrelato, em que os participantes tendem a responder conforme o comportamento socialmente esperado^(27,28)^. Além disso, é possível que a intenção declarada, mesmo em condições adversas, não se concretize na prática cotidiana, sobretudo, diante dos fatores contextuais citados, como sobrecarga de trabalho, escassez de recursos, entre outros. Deste modo, é possível afirmar que, embora haja baixa identificação das restrições das organizações como possíveis obstáculos para adesão às PP, a prática real, mais bem observada nos relatos, é influenciada por fatores organizacionais e circunstanciais, o que evidencia a necessidade de estratégias institucionais que aliem intenção, capacitação e condições estruturais favoráveis.

A análise por categorias profissionais indicou que, enquanto os técnicos de enfermagem perceberam maior “Influência social” sobre a adesão às PP, os enfermeiros sentiam maior impacto do fator “Organização”. Esse achado sugere diferentes percepções entre níveis hierárquicos. Para os técnicos, o comportamento dos pares e superiores poderia ser fator motivador ou desestimulante, evidenciando a importância das relações interpessoais para promover ambientes de adesão positiva. Para os enfermeiros, por outro lado, a estrutura institucional, que envolve ter material em todos os locais de trabalho, estar capacitado e ter acesso a formações frequentes quanto às PP, pareceu ser o principal determinante da prática, o que se coaduna com o papel gerencial que, muitas vezes, exercem.

Possível explicação para técnicos de enfermagem serem mais influenciados pelo ambiente de trabalho a aderirem às PP, em especial frente à possibilidade de advertências pelo descumprimento, reside no fato de que as atividades desses profissionais, por exigência da lei do exercício profissional de enfermagem, somente podem ser desempenhadas sob orientação e supervisão do enfermeiro^(29)^, o que pode fortalecer o papel das figuras de autoridade no reforço da adesão^(22)^.

Por outro lado, é preciso refletir que o desequilíbrio na percepção da eventualidade de advertências entre os membros da equipe de enfermagem também pode indicar cultura de vigilância punitiva, denotando ambiente organizacional mais orientado pela sanção do erro, o qual é capaz de comprometer o engajamento genuíno dos profissionais na adoção das práticas de precaução. Essa descoberta é preocupante, pois pode indicar que a cultura organizacional atual no cenário do estudo pode não estar promovendo ativamente a adesão às melhores práticas das PP^(25)^.

É importante reconhecer que adesão é uma decisão pessoal, enquanto conformidade ocorre, predominantemente, pela obediência a normas, muitas vezes, desconsiderando convicções pessoais. Desta forma, a conformidade com as PP pode ser potencializada, se forem considerados os fatores que influenciam a adesão, sobretudo aqueles relacionados ao comportamento e às atitudes da equipe de enfermagem^(27)^. Quesito que precisa ser considerado nesse âmbito é o fator “crenças pessoais”, que, neste estudo, não foi percebido pela maioria dos participantes como obstáculo à conformidade com as PP, embora a literatura demonstre que crenças individuais podem impactar diretamente a percepção pessoal do risco e, consequentemente, a extensão da proteção adotada e o comportamento de risco assumido^(13,19)^.

Nesse contexto, deve-se estimular, mais que a possibilidade de ocorrência de advertências e punições, o papel do exemplo comportamental, em que práticas positivas de adesão exercem influência direta sobre outros membros da equipe multiprofissional. Esse resultado reforça a importância da liderança pelo exemplo, com potencial para consolidar ambientes mais seguros e culturalmente positivos^(14,30)^.

No que se refere ao fator “Organização”, a capacitação quanto às PP foi destacada como facilitadora da adesão, semelhante ao encontrado em estudo realizado no Brasil, com objetivo de avaliar os fatores sociocognitivos determinantes na adesão às PP pelos profissionais de enfermagem na prática assistencial na pandemia da Covid-19^(13)^. Identifica-se que profissionais de enfermagem se beneficiam de sessões regulares de treinamento para melhorar a percepção geral sobre as medidas de PP e o controle de infecções e os riscos envolvidos na não adesão^(30)^.

Outro estudo de métodos mistos, realizado na Etiópia, com propósito de avaliar a adesão às PP para práticas de prevenção de infecções e os fatores associados entre profissionais de saúde, evidenciou que aqueles que tiveram treinamento em PP apresentaram quase quatro vezes mais probabilidade de cumpri-las, em comparação com aqueles que não fizeram treinamento^(28)^. No entanto, enfatiza-se que ações isoladas de capacitação e sensibilização não são suficientes para garantir a adesão plena às PP. É vital que as organizações de saúde ofereçam treinamento regular e as medidas necessárias para todos os enfermeiros, técnicos de enfermagem e outros profissionais de saúde para aprimorar a competência e capacidade^(26)^.

Apesar da intenção declarada de adesão ser elevada, dados qualitativos evidenciaram contradições, especialmente em contextos de urgência ou sobrecarga. Ainda assim, a intenção constitui indicador importante de potencial adesão, ao estabelecer consciência e predisposição para práticas seguras, que podem ser otimizadas, por meio de intervenções institucionais bem estruturadas, exercendo efeito mediador no desempenho relacionado ao controle de infecções, sendo determinante para práticas seguras^(13)^.

As descobertas deste estudo têm relevância significativa para atuação do profissional de enfermagem. Ao evidenciar que as restrições organizacionais, incluindo sobrecarga de trabalho, escassez de recursos e ambiente punitivo sobrepõem-se a fatores individuais, o estudo desloca o foco da responsabilização individual para necessidade de intervenções institucionais estruturais, reforçando o papel da gestão organizacional, liderança positiva e cultura de segurança. A valorização do exemplo comportamental e da liderança pelo exemplo aponta para estratégias inovadoras, baseadas em modelagem social e liderança transformacional. Desta forma, a gestão pode influenciar drasticamente os comportamentos aderentes da equipe de enfermagem, ao envolver os líderes como modelos de segurança, incentivar a comunicação aberta, reconhecer as questões relacionadas à adesão às PP e apoiar os mecanismos de segurança do paciente no local de trabalho^(27)^.

Este estudo apresenta limitações inerentes ao método adotado, que devem ser consideradas na interpretação dos resultados. Primeiramente, a natureza transversal impede a avaliação de relações causais entre as variáveis. Estudos longitudinais seriam necessários para analisar a persistência dessas intenções e a concretização ao longo do tempo. Outra limitação importante refere-se à autodeclaração dos participantes no instrumento quantitativo (SPQ) que, conforme indicado anteriormente, pode estar relacionada ao viés de desejabilidade social.

O estudo também apresenta possível limitação relacionada ao perfil da amostra, composta exclusivamente por profissionais de uma única instituição. Essa particularidade restringe a generalização dos resultados para outras realidades, uma vez que condições organizacionais, acesso a materiais, cultura de segurança e estilo de liderança podem variar consideravelmente entre diferentes instituições e regiões.

## CONCLUSÃO

O estudo mostrou que a equipe de enfermagem pesquisada possuía conhecimento satisfatório e forte intenção de aderir às precauções padrão, reconhecendo a eficácia para prevenir infecções. No entanto, persistiram desafios significativos relacionados às restrições organizacionais e à influência de fatores interpessoais.

Outrossim, destaca-se a necessidade urgente de ambientes mais colaborativos, em que a liderança positiva substitua a lógica punitiva e o exemplo de boas práticas seja valorizado. Embora a capacitação regular se mostre essencial, ela não é suficiente sem suporte estrutural adequado. Portanto, intervenções institucionais contínuas, integrando liderança positiva e melhorias organizacionais, são fundamentais para fortalecer a adesão efetiva às PP e consolidar cultura de segurança mais sólida e sustentável na prática assistencial.

## Data Availability

Todo o conjunto de dados que dá suporte aos resultados deste estudo foi publicado no próprio artigo.

# Translation: Determinantes sociocognitivos de la adherencia a las precauciones estándar por parte del personal de enfermería en un hospital regional: un estudio de métodos mixtos

**DOI:** 10.1590/1980-220X-REEUSP-2025-0335es

**Authors:** Junaiza Rodrigues Lima Nunes, Cleydson Wendel Nunes de Souza, Allyne Brena Coimbra Azevedo, Cristianne Teixeira Carneiro, Mychelangela de Assis Brito, Maria Augusta Rocha Bezerra, Ruth Cardoso Rocha

**Affiliations:** 1 Universidade Federal do Piauí, Floriano, PI, Brasil.

**Keywords:** Salud Laboral, Precauciones Universales, Enfermería

## Abstract

**Objetivo:**

Analizar los determinantes sociocognitivos de la adhesión a las precauciones estándar por parte del personal de enfermería de un hospital regional brasileño.

**Método:**

Estudio de método mixto, diseño secuencial explicativo, realizado entre enero de 2023 y marzo de 2024, con 230 profesionales. Utilizaron la versión brasileña del *Standard Precautions Questionnaire* y de las entrevistas semiestructuradas. El análisis cuantitativo empleó la prueba de Mann-Whitney y el análisis cualitativo, el análisis de contenido.

**Resultados:**

La puntuación media general de adherencia sociocognitiva fue de 3,90 (DE = 0,48), siendo la puntuación más alta en el factor “Actitud” (4,60; DE = 0,48) y la más baja en “Restricciones organizativas” (2,95; DE = 1,17). Se identificaron diferencias significativas entre las categorías profesionales en “Influencia Social” (enfermeras: 4,72; técnicos: 4,79; p = 0,001) y “Organización” (enfermeras: 4,60; técnicos: 4,49; p = 0,033). La integración de los hallazgos demostró convergencia entre los datos cuantitativos y cualitativos, mostrando que la adherencia, aunque satisfactoria, está influenciada por factores relacionados con el trabajo.

**Conclusiones:**

Los técnicos demostraron una mayor influencia social, mientras que los enfermeros identificaron más barreras organizacionales. El ejemplo de los pares fomenta la adherencia, a pesar del temor persistente a ser reprendidos por el incumplimiento de las mejores prácticas.

## INTRODUCCIÓN

Los profesionales de enfermería representan una parte significativa de la fuerza laboral en entornos hospitalarios^(1)^, estando expuestos a diferentes riesgos ocupacionales de naturaleza biológica, química, física y ergonómica^(2)^. En este contexto, los riesgos biológicos representan una de las amenazas más comunes^(3)^, ya que implican el contacto con sangre, tejidos u otros fluidos corporales potencialmente infecciosos^(2)^.

Para mitigarlos y prevenir la aparición de lesiones, es imprescindible el acceso y el cumplimiento de las medidas de bioseguridad, especialmente las Precauciones Estándar (PE), que tienen como objetivo limitar la transmisión de agentes infecciosos en los entornos de atención sanitaria^(4)^, como la higiene de las manos, el uso de equipos de protección individual (EPI), como guantes, delantales, gafas y mascarillas, y la manipulación segura de materiales punzocortantes y contaminados^(5)^.

Aunque los profesionales de enfermería muestran una mayor adhesión a las PE en comparación con otros profesionales de la salud^(6)^, los índices siguen estando por debajo de lo ideal^(7)^. Se observa que hasta el 68,3 % de los enfermeros tienen un conocimiento insatisfactorio sobre las PE y el 73,3 % no las adoptan de forma sistemática^(8)^. Las razones que se asocian con frecuencia a esta falta de adhesión incluyen la desorganización laboral, la sobrecarga de trabajo, las jornadas dobles, las situaciones de urgencia, la escasez de materiales y factores individuales, como el olvido y la baja cualificación profesional^(9)^.

Este contexto ha contribuido al aumento de la incidencia de lesiones ocupacionales entre los profesionales de enfermería^(10)^, cuya prevalencia, a lo largo de la carrera, ha alcanzado a más de la mitad de los enfermeros^(11)^. Por lo tanto, es indispensable identificar las razones subyacentes a la no adhesión a las PE^(4)^, con el fin de suplir las lagunas identificadas en estudios anteriores^(12,13)^ y profundizar en la comprensión de los determinantes que influyen en esta práctica.

Entre los factores que influyen en el comportamiento humano y la toma de decisiones de los profesionales de enfermería, destacan los determinantes sociocognitivos, como el conocimiento, la motivación, la intención, las expectativas y las percepciones^(13)^. Estos elementos desempeñan un papel fundamental en la forma en que estos profesionales se adhieren a las prácticas de PE, especialmente en un contexto caracterizado por una alta complejidad, dinamismo y situaciones a menudo urgentes^(14)^.

Sin embargo, se observa una laguna en la literatura científica en lo que respecta a los estudios que abordan específicamente los determinantes sociocognitivos y su relación con el comportamiento de los profesionales de enfermería en este escenario. Esta constatación refuerza la necesidad de una mayor comprensión, reconocimiento y evaluación crítica de estos factores^(13)^. La incorporación de datos mixtos en el análisis contribuye a una interpretación más profunda, permitiendo analizar este fenómeno de manera más amplia^(15)^. En vista de lo anterior, el objetivo fue analizar los determinantes sociocognitivos que influyen en la adhesión a las precauciones estándar por parte del equipo de enfermería en un hospital regional brasileño.

## MÉTODO

### Tipo de Estudio

El estudio utilizó un método mixto, con un diseño secuencial explicativo (QUAN→qual), en el que la fase cuantitativa precedió y orientó el análisis cualitativo. Los hallazgos cualitativos se utilizaron para profundizar en la interpretación de los resultados cuantitativos^(16)^. La etapa cuantitativa fue transversal y la cualitativa presentó un carácter analítico. Para garantizar el rigor metodológico, se aplicó la *Mixed Methods Appraisal Tool* (MMAT). La investigación buscó responder a la pregunta: ¿qué determinantes sociocognitivos influyen en la adhesión a las precauciones estándar por parte del personal de enfermería en un hospital regional brasileño?

### Lugar

La investigación se llevó a cabo en un hospital regional, ubicado en el interior del estado de Piauí, Brasil. Se trata de un hospital general, con servicios quirúrgicos, clínicos, obstétricos, pediátricos, entre otros, cuya función principal es la asistencia sanitaria. Además, la institución desarrolla actividades secundarias, como apoyo diagnóstico, atención de emergencias, dispensación de medicamentos, inmunización, promoción de la salud, prevención de enfermedades y prestación de cuidados de complejidad media y alta. En 2023, la institución contaba con 745 camas, divididas en los siguientes sectores: Centro Quirúrgico, Centro de Material y Esterilización, Clínica Quirúrgica, Clínica Médica, Obstetricia, Pediatría, Urgencias y Unidad de Cuidados Intensivos.

### Población y Criterios de Selección

La población del estudio estuvo compuesta por 377 profesionales de enfermería que trabajaban en el hospital, de los cuales 112 eran enfermeros y 265 técnicos de enfermería, según los datos proporcionados por la institución. Se establecieron como criterios de inclusión: ser profesional del equipo de enfermería que trabajara en el hospital regional desde hacía al menos un año y tener contacto directo con el paciente. Se excluyó a aquellos que estaban de vacaciones o de baja médica.

### Definición de la Muestra

La muestra se seleccionó por conveniencia, y los participantes fueron reclutados de forma presencial e individual en los diferentes sectores de la institución. Para la recolección cuantitativa, se realizó un cálculo muestral con el objetivo de garantizar un número significativo de participantes, adoptando un nivel de confianza del 95 % y un margen de error del 5 %, lo que dio como resultado una muestra mínima estimada de 191 profesionales de enfermería. Sin embargo, la muestra final se amplió, sumando un total de 230 participantes en la fase cuantitativa.

Para la recolección cualitativa, la selección de los participantes se realizó mediante muestreo intencional (*purposeful sampling*), considerando criterios de relevancia para una comprensión profunda del fenómeno investigado. Se buscó la diversidad de experiencias profesionales, cargos y sectores de actuación, a fin de garantizar la proporcionalidad entre enfermeros y técnicos de enfermería y contemplar diferentes perspectivas sobre las PE. El cierre de la muestra se produjo por saturación teórica, alcanzada cuando las nuevas entrevistas dejaron de aportar información sustancialmente nueva a las categorías analíticas, lo que dio como resultado un total de 15 participantes en esta fase.

### Recolección de Datos

La recolección de datos se llevó a cabo entre enero de 2023 y marzo de 2024. En la fase cuantitativa, se recopilaron datos de caracterización sociodemográfica (sexo, edad, etnia, ingresos) y formación profesional (categoría de formación, tiempo de formación, sector de actividad, turno de trabajo, número de empleos, satisfacción con el empleo, tiempo de trabajo en la profesión, tiempo de trabajo en la institución y enfermedad relacionada con el trabajo).

A continuación, se utilizó la versión brasileña del *Standard Precautions Questionnaire* (SPQ), desarrollado y validado originalmente en Francia, con el objetivo de evaluar los determinantes sociocognitivos en la adhesión a las PE, como actitudes, comportamientos, limitaciones personales y organizacionales^(17)^. El SPQ fue sometido a una adaptación cultural y validación semántica al portugués brasileño, presentando propiedades psicométricas satisfactorias, incluyendo validez de constructo y confiabilidad^(18)^. El instrumento se compone de 24 ítems distribuidos en siete factores: 1 – Comportamiento interpersonal (2 ítems), 2 – Restricciones organizacionales (4 ítems), 3 – Intención de seguir las PE (4 ítems), 4 – Influencia social (4 ítems), 5 – Actitudes hacia las PE (3 ítems), 6 – Organización (3 ítems) y 7 – Restricciones individuales (4 ítems)^(18,19)^.

La fase cualitativa se llevó a cabo basándose en un guion semiestructurado, elaborado para explorar la percepción de los profesionales de enfermería sobre la aplicación de las PE. Las entrevistas, con una duración media de 10 minutos, se realizaron de forma individual, en una sala reservada, se grabaron en audio y se transcribieron íntegramente. Antes de comenzar esta etapa, el guion fue validado con enfermeros de otro servicio, que no fueron incluidos en la muestra final, lo que permitió ajustar el instrumento y perfeccionar la técnica de entrevista. Cabe destacar, además, que los tres investigadores asistentes, responsables de la Recolección de datos, recibieron formación previa, orientada por la investigadora principal.

### Análisis y Tratamiento de los Datos

Los datos cuantitativos se organizaron en Microsoft Excel® 2016, mediante doble entrada, y posteriormente se analizaron en el software SPSS®, versión 20.0. Se utilizó la estadística descriptiva, incluyendo medidas de tendencia central y dispersión, así como la prueba de Shapiro-Wilk para verificar la adherencia de las variables a la distribución normal.

Al considerar la ausencia de normalidad en parte de los datos, se aplicó la prueba no paramétrica de Mann-Whitney para comparar las puntuaciones del instrumento entre las categorías profesionales^(20)^. Se adoptó un nivel de significación de p ≤ 0,05 y un intervalo de confianza del 95 %.

Los datos cualitativos se organizaron en tres etapas: preanálisis, en la que se organizó el material para hacerlo funcional y sistematizar las ideas iniciales; levantamiento/categorización del material, en la que se examinó el material y se definieron las categorías y unidades de registro y contexto. Por último, en la interpretación de los resultados, se procedió a la descripción analítica, en la que el material se sometió a un estudio en profundidad, guiado por hipótesis y referencias teóricas^(21)^. La integración de los datos se realizó mediante la conexión entre los resultados cuantitativos y cualitativos, buscando convergencias y divergencias y profundizando en el análisis del objeto de estudio.

### Aspectos Éticos

Este estudio respetó las directrices del Consejo Nacional de Salud para la investigación con seres humanos, de conformidad con las Resoluciones n.º 466/2012, 510/2016 y 580/2018, y fue aprobado por el Comité de Ética en Investigación de la Universidad Federal de Piauí, mediante los dictámenes n.º 5.834.174 y n.º 6.086.132. Se preservó la identidad de los participantes mediante la asignación de códigos alfanuméricos (P1, P2, P3…), y todos firmaron el Término de Consentimiento Libre e Ilustrado (TCLE).

## RESULTADOS

Participaron en la investigación 230 profesionales de enfermería del hospital regional, con edades comprendidas entre los 19 y los 70 años. La mayoría eran mujeres (81.7%), con predominio de jóvenes entre 19 y 40 años (80.0%).

En cuanto a la etnia, el 54,4 % se autodeclaró negro. Predominaron los técnicos de enfermería (66,1 %), con una formación de entre seis y diez años (30,4 %) y que trabajaban en los tres turnos (56,1 %). La mayoría tenía un solo vínculo laboral (68.7%) y un salario entre 1000 y 3000 reales (76.5%), además de una alta satisfacción profesional (86.5%). En cuanto al tiempo de trabajo, predominó el intervalo de uno a cinco años tanto en la profesión (43,5 %) como en la institución (47,0 %). Por último, el 75,3 % informó no presentar enfermedades ocupacionales. Los datos descritos se detallan en la Tabla 1.

**Tabla 1:** Caracterización socioeconómica y profesional de los profesionales de enfermería que trabajan en un hospital regional – PI, Brasil, 2023–2024.

Variables	N		Media ± DE
Sexo			
Femenino	188	81,7	
Masculino	42	18,3	
Edad			33,88 ± 9,75
19–40	184	80,0	
41–70	46	20,0	
Etnia			
Negro	125	54,4	
Mestizo	76	33,0	
Blanco	28	12,2	
Indígena	1	0,4	
Categoría de formación			
Técnico en enfermería	152	66,1	
Enfermero	78	33,9	
Tiempo de formación (años)			
1–5	95	41,3	
6–10	70	30,4	
> 10	64	27,9	
No informado	1	0,4	
Sector de actividad			
Centro quirúrgico	51	22,2	
Obstetricia	37	16,1	
Urgencias	31	13,5	
Clínica médica	29	12,6	
Unidad de terapia intensiva	27	11,7	
Clínica Quirúrgica	22	9,6	
Centro de Material y Esterilización	21	9,1	
Pediatría	12	5,2	
Turno de trabajo			
Mañana/Tarde/Noche	129	56,1	
Mañana	33	14,3	
Matutino/Nocturno	22	9,6	
Mañana/Tarde	15	6,5	
Nocturno	14	6,2	
Tarde	10	4,3	
Tarde/Noche	6	2,6	
No informado	1	0,4	
Número de puestos de trabajo			
Uno	158	68,7	
Dos	61	26,5	
Tres o más	9	3,9	
No informado	2	0,9	
Ingresos (salarios mínimos)			
1–3	176	76,5	
3–6	48	20,9	
> 6	5	2,2	
No informado	1	0,4	
Satisfacción con el empleo			
Sí	199	86,5	
No	31	13,5	
Tiempo de trabajo en la profesión (años)	
< 1	46	20,0	
1–5	100	43,5	
6–10	38	16,5	
> 10	45	19,6	
No informado	1	0,4	
Tiempo de trabajo en la institución (años)			
< 1	58	25,2	
1–5	108	47,0	
6–10	36	15,7	
> 10	28	12,1	
Enfermedad relacionada con el trabajo			
Sí	47	20,4	
No	173	75,3	
No sé	10	4,3	

En la Tabla 2 se presentan los resultados obtenidos con la aplicación del cuestionario *Standard Precautions Questionnaire* (SPQ). En el factor “Actitud” (parte 1), la mayoría de los participantes estuvieron totalmente de acuerdo en que las PE constituyen una forma eficaz de prevenir las infecciones hospitalarias (68,3 %), así como de proteger a los pacientes (67,4 %) y a los profesionales (67,4 %). En el factor “Influencia social”, el 36,1 % creía parcialmente que sus compañeros valoraban las PE. Sin embargo, el 48,7 % mencionó el riesgo de recibir una advertencia de sus superiores cuando no las seguían, siendo el porcentaje más prevalente en este ámbito. En cuanto al factor “Organización”, destacaron tres ítems con una prevalencia superior al 60 %: el 65,7 % informó de la total eficacia de la disponibilidad de material; el 60,4 % indicó que estar capacitado favorece la adhesión; y el 61,3 % reforzó la importancia de las capacitaciones frecuentes.

**Tabla 2:** Distribución, porcentaje, media y desviación estándar, según los ítems de la versión brasileña del Cuestionario de Precauciones Estándar (*Standard Precautions Questionnaire*), respondidos por los profesionales de enfermería – PI, Brasil, 2023–2024.

Ítems	Nada eficaz n (%)	Algo eficaz n (%)	Más o menos eficaz n (%)	Parcialmente eficaz n (%)	Totalmente eficaz n (%)	No respondió n (%)	Media	Desviación estándar
**Parte 1: Actitud**								
Las precauciones estándar son medidas eficaces para reducir las infecciones hospitalarias	5 2,2%	–	8 3,5%	59 25,7%	157 68,3%	1 0,4%	4,59	0,76
Si sigo las medidas de precaución estándar, protegeré a mis pacientes de una infección.	–	–	9 3,9%	65 28,3%	155 67,4%	1 0,4%	4,64	0,56
Seguir las medidas de precaución estándar me protegerá de una infección.	1 0,4%	2 0,9%	11 4,8%	62 27%	154 67,0%	–	4,59	0,66
**Parte 2 – Influencia social**								
La mayoría de mis compañeros de trabajo piensan que es importante seguir las precauciones estándar	4 1,7%	14 6,1%	70 30,4%	83 36,1%	57 24,8%	2 0,9%	3,78	0,97
Corro el riesgo de recibir advertencias de mis superiores si no sigo las precauciones estándar	5 2,2%	8 3,5%	22 9,6%	83 36,1%	112 48,7%	–	4,25	0,92
Corro el riesgo de recibir advertencias de los enfermeros y auxiliares responsables de la higiene si no sigo las precauciones estándar.	8 3,5%	16 7,0%	27 11,7%	87 37,8%	90 39,1%	1 0,4%	4,03	1,06
Corro el riesgo de recibir advertencias de los médicos si no sigo las precauciones estándar.	19 8,3%	19 8,3%	38 16,5%	69 30,0%	83 36,1%	2 0,9%	3,80	1,26
**Parte 3 – Organización**								
Disponer de material (calidad, disponibilidad y accesibilidad) en todos los lugares de trabajo	1 0,4%	4 1,7%	19 8,3%	54 23,5%	151 65,7%	1 0,4%	4,53	0,76
Estar capacitado en lo que se refiere a las precauciones estándar	1 0,4%	2 0,9%	16 7,0%	70 30,4%	139 60,4%	2 0,9%	4,52	0,71
Tener formación sobre las precauciones estándar	2 0,9%	2 0,9%	10 4,3%	74 32,2%	141 61,3%	1 0,4%	4,53	0,70
**Parte 4 – Comportamiento interpersonal**								
Cuando el profesional médico tiene un comportamiento ejemplar en relación con las precauciones estándar	3 1,3%	14 6,1%	28 12,2%	68 29,6%	116 50,4%	1 0,4%	4,23	0,97
Cuando mis compañeros de trabajo tienen un comportamiento ejemplar en relación con las precauciones estándar	8 3,5%	7 3,0%	30 13,0%	76 33,0%	108 47,0%	1 0,4%	4,18	1,01
**Elementos**	**No es un obstáculo n (%)**	**Un poco obstáculo n (%)**	**Más o menos obstáculo n (%)**	**Obstáculo obstáculo n (%)**	**Es un obstáculo n (%)**	**No respondió n (%)**	**Media**	**Desviación estándar**
**Parte 5 – Restricciones organizativas**								
Situaciones inesperadas que pueden interferir en la realización de mi trabajo (urgencias, peticiones de compañeros, nuevas tareas que cumplir).	44 19,1%	16 7,0%	60 26,1%	52 22,6%	56 24,3%	2 0,9%	3,28	1,42
Falta de tiempo.	89 38,7%	32 13,9%	36 15,7%	30 13,0%	41 17,8%	2 0,9%	2,60	1,56
Carga de trabajo más elevada de lo habitual.	56 24,3%	31 13,5%	52 22,6%	30 13,0%	58 25,2%	3 1,3%	3,05	1,54
Complejidad de las medidas de precaución estándar.	66 28,7%	32 13,9%	50 21,7%	33 14,3%	46 20,0%	3 1,3%	2,87	1,53
**Parte 6 – Restricciones individuales**								
Desconocimiento de las precauciones estándar.	55 23,9%	21 9,1%	33 14,3%	27 11,7%	91 39,6%	3 1,3%	3,37	1,65
Rutina, hábitos y equipo de trabajo.	81 35,2%	22 9,6%	40 17,4%	28 12,2%	57 24,8%	2 0,9%	2,84	1,63
Creencias personales relacionadas con las precauciones estándar.	88 38,3%	21 9,1%	42 18,3%	24 10,4%	52 22,6%	3 1,3%	2,73	1,64
Problemas relacionados con el material (calidad, disponibilidad y accesibilidad).	35 15,2%	15 6,5%	37 16,1%	44 19,1%	97 42,2%	2 0,9%	3,69	1,47
**Elementos**	**Nunca n (%)**	**Rara vez n (%)**	**A veces n (%)**	**Muchas veces n (%)**	**Siempre n (%)**	**No respondió n (%)**	**Media**	**Desviación estándar**
**Parte 7 – Intención de seguir las precauciones estándar**								
Incluso cuando el paciente sea difícil.	3 1,3%	6 2,6%	24 10,4%	29 12,6%	167 72,6%	1 0,4%	4,53	0,88
Incluso cuando hay poco tiempo.	2 0,9%	11 4,8%	25 10,9%	39 17,0%	152 66,1%	1 0,4%	4,43	0,93
Incluso cuando me duelen o me lastimo las manos.	18 7,8%	7 3,0%	22 9,6%	44 19,1%	139 60,4%	–	4,21	1,21
Incluso en situaciones de urgencia	5 2,2%	10 4,3%	21 9,1%	26 11,3%	167 72,6%	1 0,4%	4,49	0,98

En “Comportamiento interpersonal”, el 50,4 % señaló que el comportamiento ejemplar de los médicos facilita la adhesión y el 47,0 % reforzó la influencia positiva de los compañeros. En cuanto a las “Restricciones organizativas”, el principal obstáculo fueron las “situaciones inesperadas” (24,3 %), mientras que la “falta de tiempo” no se consideró un obstáculo para el 38,7 %. En “Restricciones individuales”, el mayor obstáculo e fue el material inadecuado (42,2 %), mientras que las creencias personales se minimizaron (el 38,3 % declaró que no eran obstáculos). Por último, en “Intención”, la mayoría siempre seguía las PE, especialmente en situaciones difíciles (72,6 %).

La escala SPQ presentó una media general de 3,90 (DE = 0,48). El factor “Actitud” obtuvo la puntuación más alta (4,60; DE: 0,48), mientras que el factor “Restricciones organizativas” registró la media más baja (2,95; DE: 1,17). En la comparación por categoría profesional (Tabla 3), los técnicos de enfermería obtuvieron una puntuación significativamente mayor en “Influencia social” (4,09) en relación con los e enfermeros (3,72; p = 0,001), mientras que los enfermeros obtuvieron una puntuación más alta en el factor “Organización” (4,60) en comparación con los técnicos (4,49; p = 0,033). En cuanto a los factores “Actitud”, “Comportamiento interpersonal”, “Restricciones organizativas”, “Restricciones individuales” e “Intención de seguir las precauciones estándar”, no se observaron diferencias estadísticas relevantes entre los grupos.

**Tabla 3:** Puntuación de los determinantes sociocognitivos relativos a los factores de la versión brasileña del *Standard Precautions Questionnaire* (*Cuestionario de Precauciones Estándar*) por categoría profesional de los profesionales de enfermería – PI, Brasil, 2023–2024.

Factores	Categorías profesionales		Valor de p*
	Enfermero	Técnico de enfermería	
Actitud	4,64	4,58	0,293
Influencia social	3,72	4,09	**0,001**
Organización	4,60	4,49	**0,033**
Comportamiento interpersonal	4,07	4,27	0,331
Restricciones organizativas	3,06	2,89	0,250
Restricciones individuales	3,14	3,17	0,947
Intención de seguir las precauciones estándar	4,42	4,41	0,870

*Prueba de Mann-Whitney.

En cuanto al análisis cualitativo del estudio, el contenido de las entrevistas permitió establecer tres categorías analíticas: comprensión de las precauciones estándar por parte del personal de enfermería, uso de las precauciones estándar por parte del personal de enfermería y factores que dificultan el uso de las precauciones estándar por parte del personal de enfermería. Estas categorías se incorporaron a los resultados cuantitativos, extrayendo declaraciones significativas para representarlos mejor, tal y como se describe en la presentación conjunta que se muestra en la Cuadro 1.

**Cuadro 1:** Visualización conjunta con integración de los resultados cuantitativos y las declaraciones de los participantes – PI, Brasil, 2023 – 2024.

Subcategoría	Resultados cuantitativos (n = 230)	Resultados cualitativos (n = 15)
**Categoría 1 – Comprensión de los profesionales de enfermería sobre las precauciones estándar**		
Comprensión del concepto de las PE.	El 68,3 % indicó que las PE son medidas eficaces para reducir las infecciones hospitalarias. El 39,6 % indicó que la falta de conocimiento sobre las PE es un obstáculo. El 38,3 % indicó que las creencias personales relacionadas con las PE no son un obstáculo para su cumplimiento. El 60,4 % indicó que estar capacitado en materia de PE es totalmente eficaz para la adhesión. El 61,3 % indicó que tener formación en PE es totalmente eficaz para la adhesión.	*La precaución proviene de la prevención, la profilaxis y la prevención, y la norma es porque es una forma ya preparada para ser seguida, por lo que el objetivo de la precaución estándar es buscar estandarizar las formas que deben seguir todos los profesionales de la salud, con el objetivo de prevenir o incluso controlar la transmisión de microorganismos en el entorno laboral, principalmente en el ámbito hospitalario*. (P13)
Indicación sobre las principales medidas de PE utilizadas.	El 65,7 % indicó que disponer de material (calidad, disponibilidad y accesibilidad) en todos los lugares de trabajo es eficaz. El 42,2 % indicó que los problemas relacionados con el material (calidad, disponibilidad y accesibilidad) son obstáculos para el cumplimiento de las PE.	*Equipos de protección individual, gafas, mascarillas, lavado correcto de manos, higienización del entorno crítico, por ejemplo, como el del centro quirúrgico, de equipos, colocar los materiales punzantes en el lugar adecuado y todo ello evita posibles accidentes laborales y/o alguna contaminación*. (P1)
Percepción de que el uso de las PE previene accidentes y protege al profesional y al paciente contra enfermedades.	El 67,4 % indicó que seguir las medidas de PE protegerá a los pacientes de una infección. El 67,0 % indicó que seguir las medidas de PE protegerá al profesional de una infección.	*Las PE previenen problemas o cualquier tipo de infección tanto del paciente al profesional como del profesional al paciente*. (P1)
**Categoría 2: Uso de las precauciones estándar por parte del personal de enfermería**		
Cuándo deben utilizarse las PE.	El 72,6 % indicó que siempre tiene la intención de seguir las PE, incluso cuando el paciente es difícil. El 66,1 % indicó que siempre tiene la intención de seguir las PE, incluso cuando hay poco tiempo. El 60,4 % indicó que siempre tiene la intención de seguir las PE, incluso cuando le duelen o le sangran las manos. El 72,6 % indicó que siempre tiene la intención de seguir las PE, incluso en situaciones de urgencia. El 38,7 % indicó que la falta de tiempo no es un obstáculo para cumplir las PE.	*[…] deben utilizarse siempre en la asistencia a un paciente y a otro […] ¿habrá excepciones? ¡Sí! Dependiendo del caso, pero se recomienda utilizarlas siempre*. (P13) *[…] en caso de emergencia, digamos que hay una parada cardiorrespiratoria: estás realizando un procedimiento sencillo y s que reanimar y no hay tiempo para ponerse todo el equipo, pero entonces usamos lo que tenemos a mano*. (P12)
**Categoría 3: Factores que interfieren en el uso de las precauciones estándar por parte del personal de enfermería**		
Negligencia, olvido, falta de cuidado, descuido y alergia.	El 42,2 % indicó que los problemas relacionados con el material (calidad, disponibilidad y accesibilidad) son obstáculos para el cumplimiento de las PE. El 25,2 % indicó que una carga de trabajo más elevada de lo habitual es un obstáculo para el cumplimiento de las PE.	*De cualquier manera, es negligencia: hay que vigilarse todo el tiempo para estar siempre dentro de los estándares*. (P7) *Aunque sé lo importante que es, a veces no lo uso porque no tengo tiempo; a veces, uno se relaja […] y confía, aunque sabe que “quien ve la cara, no ve la enfermedad”; y, a veces, ni siquiera lo uso tanto porque soy alérgica al látex*. (P5) *Cuando la institución no proporciona EPI, cuando hay falta o no se ha repuesto […]*. (P13) *¿Quiere que sea sincero? Bueno, siendo muy sincero, cuando no uso las precauciones estándar es por falta de cuidado, solo por eso, aun sabiendo que protegen a los profesionales y a los pacientes*. (P15)
Falta de disponibilidad de materiales.	El 65,7 % indicó que disponer de material (calidad, disponibilidad y accesibilidad) en todos los lugares de trabajo es totalmente eficaz para el cumplimiento de las PE.	*[…] dejar de usarlos porque queremos, no lo hacemos, solo dejamos de usarlos cuando no los tenemos. Y es difícil que falten, pero a veces faltan y, cuando faltan, improvisamos*. (P1)
Influencia colectiva.	El 36,1 % indicó que es parcialmente eficaz cuando la mayoría de los compañeros de trabajo piensan que es importante seguir las PE. El 50,4 % indicó que es totalmente eficaz cuando el profesional médico tiene un comportamiento ejemplar en relación con las PE. El 47,0 % indicó que es totalmente eficaz cuando los compañeros de trabajo tienen un comportamiento ejemplar en relación con las PE.	*[…] porque cuando uso mi EPI, estoy segura: no voy a contagiarme ni mucho menos transmitir el virus. Si todo el mundo tomara las medidas de precaución, sería más interesante, pero no todo el mundo las toma*. (P9)

**Nota:** PE = precauciones estándar.

La integración de los resultados evidencia una fuerte convergencia entre las dimensiones evaluadas, sobre todo en el reconocimiento de la importancia de las PE y en la percepción de su eficacia para prevenir infecciones, aspecto corroborado tanto por las altas puntuaciones de “Actitud” en el componente cuantitativo como por los informes que destacaron el carácter preventivo y la protección mutua que proporcionan las medidas. También se observó convergencia en la identificación de barreras organizativas, especialmente en lo que se refiere a la disponibilidad y adecuación de los materiales, señalada cuantitativamente como el principal obstáculo y reiterada cualitativamente por la mención a la falta de EPI y la necesidad de improvisar. La influencia colectiva también se mostró consistente entre los métodos: aunque el análisis cuantitativo indicó una influencia social moderada, los discursos reforzaron que el comportamiento ejemplar de los compañeros y médicos favorece la adhesión.

Por otro lado, surgieron sutiles divergencias en cuanto al uso efectivo de las PE: mientras que los datos cuantitativos sugirieron una alta intención de adhesión, incluso en situaciones adversas, los testimonios cualitativos revelaron situaciones de negligencia, olvido e improvisación, lo que indica un desajuste entre la intención declarada y la práctica cotidiana. En general, la convergencia de los hallazgos sostiene que el conocimiento, la disponibilidad de recursos y el entorno colectivo constituyeron determinantes centrales de la adhesión, mientras que las divergencias destacaron la necesidad de estrategias institucionales orientadas a la reducción de barreras prácticas y conductuales.

## DISCUSIÓN

El análisis de los determinantes sociocognitivos del equipo de enfermería de un hospital regional brasileño evidenció avances importantes en el conocimiento y la intención declarada de los enfermeros y técnicos de enfermería en cuanto a la adhesión a las PE, especialmente en lo que respecta al reconocimiento de la eficacia en la prevención de infecciones hospitalarias. Sin embargo, esta percepción positiva contrasta con las barreras prácticas identificadas, particularmente en lo que respecta a las restricciones organizativas y la disponibilidad de materiales, lo que indica la persistencia del conocido desajuste entre el saber y el hacer en la práctica clínica. Estudios anteriores demostraron que el nivel de conocimiento de los profesionales de enfermería sobre las PE era satisfactorio^(22,23)^.

Sin embargo, esta práctica aún no ha sido incorporada por la mayoría^(22)^, especialmente debido a la baja calidad, disponibilidad y accesibilidad de los materiales, lo que constituye un importante obstáculo para su adopción^(24)^. La traducción de los conceptos de PE a la práctica es, por lo tanto, un reto, y se necesita el apoyo de la institución sanitaria para promover la adhesión a las normas por parte de todo el equipo^(25)^.

Los determinantes sociocognitivos relacionados con la “restricción organizativa” obtuvieron la puntuación media más baja en la escala global, lo que indica que los profesionales de enfermería no consideraron que las situaciones inesperadas (por ejemplo, las urgencias), la escasa disponibilidad de tiempo, la mayor carga de trabajo y la complejidad de las medidas de PE constituyeran obstáculos para la adhesión. Este resultado es preocupante, ya que estas limitaciones institucionales siguen siendo uno de los mayores obstáculos prácticos para el uso sistemático de las PE, superando a menudo a factores individuales, como el desconocimiento o las creencias personales.

Un estudio realizado en Arabia Saudita con el objetivo de investigar el cumplimiento de las PE entre enfermeros y médicos identificó que el principal reto que afectaba a la adherencia de los enfermeros a las PE era el exceso de carga de trabajo (82,1 %)^(26)^. Una revisión de alcance realizada con el objetivo de identificar los factores comunes relacionados con la falta de adhesión de los enfermeros a las prácticas de prevención y control de infecciones estableció que el tiempo era un recurso de particular importancia en relación con la adhesión a las PE, relacionado principalmente con el tiempo dedicado a ponerse el EPI. Además, se destacó que, en situaciones de urgencia, los enfermeros informaron que a menudo priorizan la atención de emergencia por encima de las precauciones de prevención y control de infecciones^(24)^.

Esta divergencia se identificó en los datos de la etapa cuantitativa y la literatura también se verificó en el análisis cualitativo, con los participantes indicando excepciones para no usar las PE, como la falta de tiempo y las situaciones de urgencia.

Esta incongruencia puede explicarse en parte por el sesgo de deseabilidad social, común en los estudios de autoinforme, en los que los participantes tienden a responder de acuerdo con el comportamiento socialmente esperado^(27,28)^. Además, es posible que la intención declarada, incluso en condiciones adversas, no se materialice en la práctica cotidiana, sobre todo ante los factores contextuales mencionados, como la sobrecarga de trabajo, la escasez de recursos, entre otros. De este modo, se puede afirmar que, aunque existe una baja identificación de las restricciones de las organizaciones como posibles obstáculos para la adhesión a las PE, la práctica real, más bien observada en los relatos, está influenciada por factores organizacionales y circunstanciales, lo que evidencia la necesidad de estrategias institucionales que combinen intención, capacitación y condiciones estructurales favorables.

El análisis por categorías profesionales indicó que, mientras que los técnicos de enfermería percibían una mayor “influencia social” sobre la adhesión a las PE, los enfermeros sentían un mayor impacto del factor “organización”. Este hallazgo sugiere diferentes percepciones entre los niveles jerárquicos. Para los técnicos, el comportamiento de sus compañeros y superiores podía ser un factor motivador o desmotivador, lo que pone de manifiesto la importancia de las relaciones interpersonales para promover entornos de adhesión positiva. Para los enfermeros, por otro lado, la estructura institucional, que implica disponer de material en todos los lugares de trabajo, estar capacitado y tener acceso a formaciones frecuentes sobre las PE, pareció ser el principal determinante de la práctica, lo que concuerda con el papel gerencial que, a menudo, ejercen.

Una posible explicación de que los técnicos de enfermería se vean más influenciados por el entorno de trabajo para adherirse a las PE, especialmente ante la posibilidad de recibir advertencias por incumplimiento, reside en el hecho de que las actividades de estos profesionales, por exigencia de la ley del ejercicio profesional de la enfermería, solo pueden realizarse bajo la orientación y supervisión del enfermero^(29)^, lo que puede fortalecer el papel de las figuras de autoridad en el refuerzo de la adhesión^(22)^.

Por otro lado, hay que reflexionar sobre el hecho de que el desequilibrio en la percepción de la posibilidad de recibir advertencias entre los miembros del equipo de enfermería también puede indicar una cultura de vigilancia punitiva, lo que denota un entorno organizativo más orientado a sancionar los errores, lo que puede comprometer el compromiso genuino de los profesionales con la adopción de prácticas de precaución. Este hallazgo es preocupante, ya que puede indicar que la cultura organizacional actual en el escenario del estudio puede no estar promoviendo activamente la adhesión a las mejores prácticas de las PE^(25)^.

Es importante reconocer que la adhesión es una decisión personal, mientras que el cumplimiento se produce, predominantemente, por la obediencia a las normas, a menudo sin tener en cuenta las convicciones personales. De este modo, el cumplimiento de las PE puede potenciarse si se tienen en cuenta los factores que influyen en la adhesión, sobre todo los relacionados con el comportamiento y las actitudes del equipo de enfermería^(27)^. Un aspecto que debe tenerse en cuenta en este ámbito es el factor “creencias personales”, que, en este estudio, no fue percibido por la mayoría de los participantes como un obstáculo para el cumplimiento de las PE, aunque la literatura demuestra que las creencias individuales pueden influir directamente en la percepción personal del riesgo y, en consecuencia, en el alcance de la protección adoptada y el comportamiento de riesgo asumido^(13,19)^.

En este contexto, más que la posibilidad de advertencias y sanciones, se debe fomentar el papel del ejemplo conductual, en el que las prácticas positivas de adhesión ejercen una influencia directa sobre otros miembros del equipo multidisciplinario. Este resultado refuerza la importancia del liderazgo por ejemplo, con potencial para consolidar entornos más seguros y culturalmente positivos^(14,30)^.

En lo que respecta al factor “Organización”, la capacitación en materia de PE se destacó como facilitadora de la adhesión, de manera similar a lo encontrado en un estudio realizado en Brasil, con el objetivo de evaluar los factores sociocognitivos determinantes en la adhesión a las PE por parte de los profesionales de enfermería en la práctica asistencial durante la pandemia de Covid-19^(13)^. Se identifica que los profesionales de enfermería se benefician de sesiones regulares de capacitación para mejorar la percepción general sobre las medidas de PE y el control de infecciones y los riesgos que implica la no adhesión^(30)^.

Otro estudio de métodos mixtos, realizado en Etiopía con el propósito de evaluar la adhesión a las PE para prácticas de prevención de infecciones y los factores asociados entre los profesionales de la salud, evidenció que aquellos que recibieron capacitación en PE tenían casi cuatro veces más probabilidades de cumplirlas, en comparación con aquellos que no recibieron capacitación^(28)^. Sin embargo, se hace hincapié en que las acciones aisladas de capacitación y sensibilización no son suficientes para garantizar el pleno cumplimiento de las PE. Es fundamental que las organizaciones de salud ofrezcan capacitación regular y las medidas necesarias a todos los enfermeros, técnicos de enfermería y otros profesionales de la salud para mejorar su competencia y capacidad^(26)^.

A pesar de que la intención declarada de adherirse es elevada, los datos cualitativos han puesto de manifiesto contradicciones, especialmente en contextos de urgencia o sobrecarga. Aun así, la intención constituye un indicador importante de la adhesión potencial, al establecer la conciencia y la predisposición hacia prácticas seguras, que pueden optimizarse mediante intervenciones institucionales bien estructuradas, ejerciendo un efecto mediador en el desempeño relacionado con el control de infecciones, lo que es determinante para las prácticas seguras^(13)^.

Los hallazgos de este estudio tienen una relevancia significativa para la actuación del profesional de enfermería. Al evidenciar que las restricciones organizativas, incluyendo la sobrecarga de trabajo, la escasez de recursos y el ambiente punitivo se superponen a los factores individuales, el estudio desplaza el foco de la responsabilidad individual hacia la necesidad de intervenciones institucionales estructurales, reforzando el papel de la gestión organizativa, el liderazgo positivo y la cultura de seguridad. La valoración del ejemplo conductual y del liderazgo por el ejemplo apunta a estrategias innovadoras, basadas en el modelado social y el liderazgo transformacional. De esta manera, la gestión puede influir drásticamente en los comportamientos adherentes del equipo de enfermería, al involucrar a los líderes como modelos de seguridad, fomentar la comunicación abierta, reconocer las cuestiones relacionadas con la adhesión a las PE y apoyar los mecanismos de seguridad del paciente en el lugar de trabajo^(27)^.

Este estudio presenta limitaciones inherentes al método adoptado, que deben tenerse en cuenta en la interpretación de los resultados. En primer lugar, la naturaleza transversal impide la evaluación de las relaciones causales entre las variables. Se necesitarían estudios longitudinales para analizar la persistencia de estas intenciones y su materialización a lo largo del tiempo. Otra limitación importante se refiere a la autodeclaración de los participantes en el instrumento cuantitativo (SPQ) que, como se ha indicado anteriormente, puede estar relacionada con el sesgo de deseabilidad social.

El estudio también presenta una posible limitación relacionada con el perfil de la muestra, compuesta exclusivamente por profesionales de una sola institución. Esta particularidad limita la generalización de los resultados a otras realidades, ya que las condiciones organizativas, el acceso a los materiales, la cultura de seguridad y el estilo de liderazgo pueden variar considerablemente entre diferentes instituciones y regiones.

## CONCLUSIÓN

El estudio mostró que el personal de enfermería encuestado tenía un conocimiento satisfactorio y una fuerte intención de adherirse a las Precauciones estándar, reconociendo su eficacia para prevenir infecciones.

Sin embargo, persistieron desafíos significativos relacionados con las restricciones organizativas y la influencia de factores interpersonales.

Asimismo, se destaca la necesidad urgente de entornos más colaborativos, en los que el liderazgo positivo sustituya a la lógica punitiva y se valore el ejemplo de las buenas prácticas. Aunque la capacitación regular resulta esencial, no es suficiente sin un apoyo estructural adecuado. Por lo tanto, las intervenciones institucionales continuas, que integren el liderazgo positivo y las mejoras organizativas, son fundamentales para fortalecer la adhesión efectiva a las PE y consolidar una cultura de seguridad más sólida y sostenible en la práctica asistencial.

## Data Availability

Todo el conjunto de datos que apova los resultados de este estudio fue publicado en el propio artículo.
